# Generative Artificial Intelligence-driven orthodontic education practices

**DOI:** 10.1186/s12909-026-09276-7

**Published:** 2026-04-29

**Authors:** Menghan Zhang, Yuzhi Yang, Yan lv, Yanfang Yu, Sihui Hu, Ziyuan Yang, Zhiwei Wang, Mengjie Wu

**Affiliations:** 1 Stomatology Hospital, School of Stomatology, Zhejiang Provincial Clinical Research Center for Oral Diseases, Zhejiang University School of Medicine, Hangzhou, 310005 China; 2 Key Laboratory of Oral Biomedical Research of Zhejiang Province, Engineering Research Center of Oral Biomaterials and Devices of Zhejiang Province, Cancer Center of Zhejiang University, Hangzhou, 310005 China

**Keywords:** Generative Artificial Intelligence, Orthodontics education

## Abstract

Generative Artificial Intelligence (GenAI) is transforming medical education, including in the field of orthodontics. This paper presents an overview of prominent GenAI models and orthodontic platforms, such as ChatGPT, DeepSeek, GANs, and Diffusion Models, CephGPT-4, iOrthoPredictor, offer a wide range of capabilities, from knowledge-based Q&A and clinical diagnostics to image generation and treatment simulation. The integration of GenAI with established teaching strategies and learning theories were also introduced, with exmples exploring GenAI applications across four key domains of orthodontic education: knowledge dissemination, clinical practice, teaching outcome assessment, and medical research. GenAI demonstrates capabilities in generating personalized educational content, optimizing curriculum design, and enhancing learning efficiency, and facilitates case simulation, diagnostic assistance, and virtual training modules, thereby supporting the development of practical clinical skills. The technology further contributes to education through personalized performance assessments and feedback mechanisms that improve learning outcomes. In research area, GenAI aids in literature retrieval, data analysis, and academic writing. Despite these promising applications, limitations such as inaccurate information, ethical challenges, excessive dependence, academic misconduct and educational integrity also exist. Proposed solutions involve the integration of GenAI with validated medical resources, implementation of robust data security protocols, and the establishment of guidelines for responsible utilization in educational settings. While GenAI offers significant potential to advance orthodontic education, its effective and ethical implementation requires careful navigation of these limitations and challenges. The development of appropriate safeguards and best practice guidelines will be essential to maximize benefits while mitigating risks associated with this emerging technology.

**Supplementary Information:**

The online version contains supplementary material available at 10.1186/s12909-026-09276-7.

## Background

Orthodontics is a branch of dentistry that emphasizes diagnosing, preventing, and managing craniofacial problems and malocclusions, with a variety of fixed and removable orthodontic appliances. The field presents significant educational challenges due to its highly specialized nature and prolonged treatment duration (typically approximately two years), making comprehensive mastery difficult during undergraduate dental training. Consequently, most orthodontic instruction occurs within dedicated postgraduate programs or continuing education courses. Internationally, formal postgraduate orthodontic training programs generally require 2–4 years of study resulting in substantial educational investments [1]. This extended training requirement, combined with the current global shortage and uneven geographical distribution of orthodontic specialists has created a significant disparity between provider availability and the growing demand for orthodontic care among both adolescent and adult populations [2, 3].

Generative Artificial Intelligence (GenAI) refers to Artificial Intelligence systems capable of synthesizing novel content based on input data. Unlike conventional AI systems that primarily analyze, predict, reason, or classify existing data, GenAI extends its functionality to the creation of original content, including text, images, and audio. To date, this technology has found widespread application across diverse domains [4]. In medical education, GenAI integrates medical knowledge, professional databases and medical imaging to facilitate students’ comprehension of foundational concepts [5]. Furthermore, GenAI can provide constructive feedback, thereby fostering the development of clinical reasoning and practical competencies [6].

Currently, GenAI is employed in orthodontics to support data-driven, rule-based decision-making and to analyze clinical imagery, intraoral scans, and photographic datasets [7, 8]. Although GenAI has been applied in designing educational strategies for orthodontic training [9, 10], its use remains at an exploratory stage. Nevertheless, its integration into orthodontic education holds substantial developmental potential, as it may enhance instructional quality and efficiency while fostering the training of skilled orthodontic professionals [11, 12]. This paper introduces common GenAI models and platforms, examines their current landscape, explores their potential applications in orthodontic education, illustrates specific teaching scenarios, and identifies key challenges in implementation, with the aim of providing insights and strategies to optimize the integration of GenAI in orthodontic education.

## Methods

A comprehensive literature search was conducted to identify relevant studies. The search covered articles published between January 2020 and September 2025. This timeframe was selected to capture the most recent advancements in GenAI and its applications in orthodontic education. The inclusion of studies focusing on GenAI developments post-2020 recognizes that early models such as GPT-3.5, GPT-4, and Google Bard, contributed significantly to the evolution of AI-driven educational tools.

The search included databases such as PubMed, ERIC, and IEEE Xplore, using three categories of keywords: (1) “Orthodontic”, “Malocclusion” et al.; (2) “Generative Artificial Intelligence”, “Artificial Intelligence”, “ChatGPTs”, “Deepseek”, “Bard”, “ Gemini”, “ Bing Chat” “GANs”“ Diffusion”“Transformer”, et al.; (3) “Education”, “Teaching”, “Student”, “Exam”, “Question”, et al. In addition to database searches, a manual search was conducted by reviewing the reference lists of relevant articles. After removing duplicate results, the retrieved papers were screened based on their titles and abstracts. To be included in the review, studies had to primarily focus on GenAI being utilized or showing potential for orthodontics education. Eligible study designs included methodological research (e.g., algorithm and model development, educational methodology) and experimental methodological research (randomized controlled trials, cohort studies, cross-sectional studies, and qualitative research). The full texts of the remaining papers were then reviewed. Studies that were not available in full text, those focusing on general AI applications without specific relevance to orthodontic education, and dental education studies that did not include an orthodontic component were excluded. Two independent reviewers conducted the search and retrieval process to ensure accuracy and reduce bias. Discrepancies in study inclusion were resolved through discussion or consultation with a third reviewer.

A customized checklist was used to assess the levels of evidence of the cited studies, as previously described [13, 14]. Each criterion was scored as 2 (fully met), 1 (partially met or with certain limitations), or 0 (not met). The overall quality of each study was then categorized as follows: (i) strong (total score 80%–100%), (ii) moderate (65%–79%), or (iii) weak (≤ 64%). Studies were categorized based on total scores as: (i) strong (80%-100%), (ii) moderate (65%-79%), or (iii) weak (≤ 64%). Two authors independently evaluated all studies, resolving disagreements through re-assessment until consensus was reached. The complete quality assessment data, including the defined criteria, results for each study, and their evidence levels, are presented in Table S1.

## GenAI models and platforms in orthodontics

**Fig. 1:**
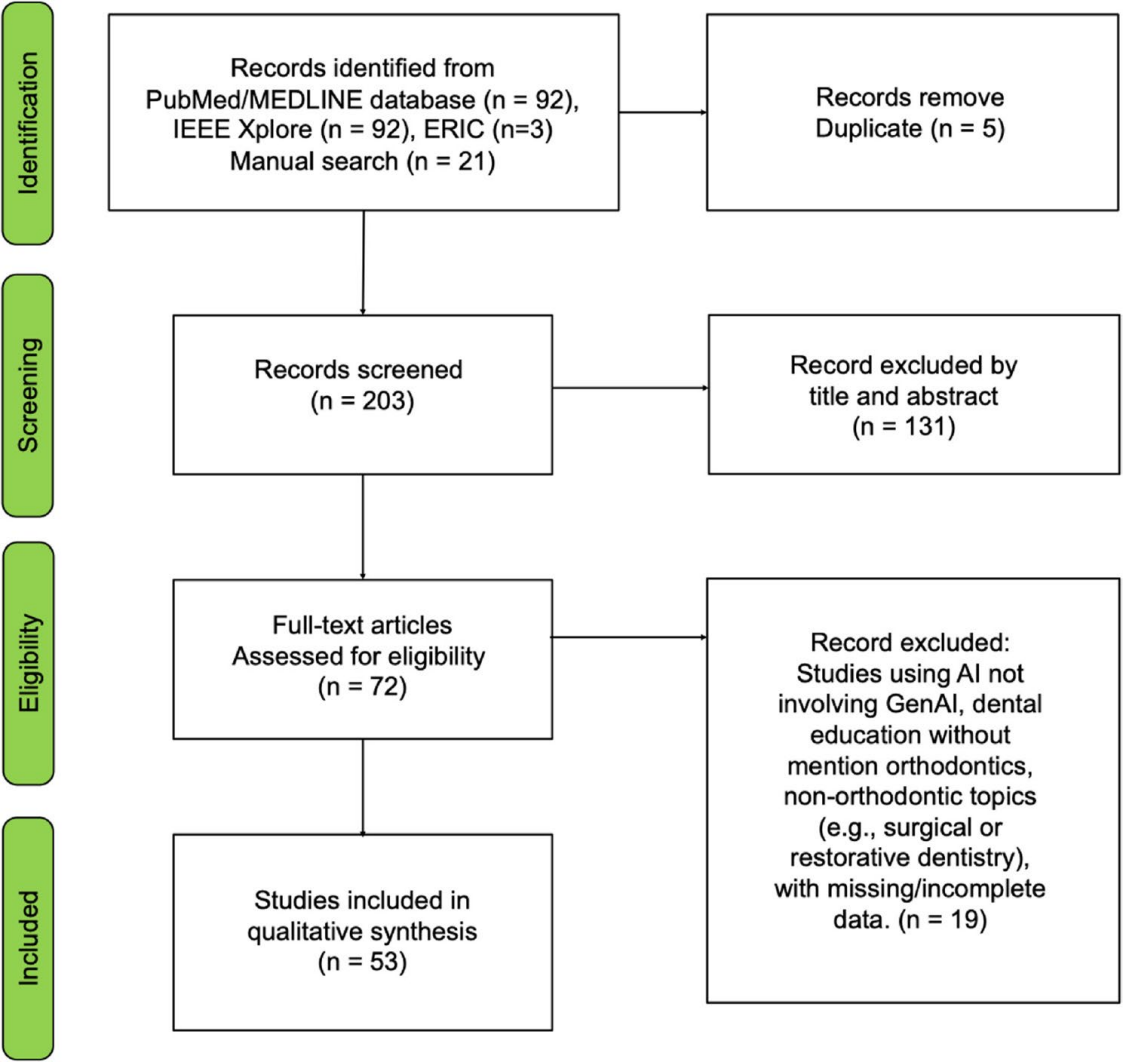
A flowchart illustrating the article screening process

The rapid evolution of GenAI has introduced transformative tools for medical and orthodontic applications. The selection process is illustrated in Fig. 1, and finally 53 articles were selected for synthesis after applying the eligibility criteria. Prominent GenAI models such as ChatGPT, DeepSeek, Generative Adversarial Networks (GANs), and Diffusion Models exhibit a wide range of capabilities, spanning from knowledge-based Question and Answer (Q&A) and clinical diagnostics [15–52] (Tables 1 and 2) to image generation and treatment simulations [53–60] (Tables 3 and 4), supporting applications in both clinical decision-making and educational innovation. Orthodontic-specific platforms like iOrtho, CephGPT-4, and iOrthoPredictor offer specialized services, including automated cephalometric analysis, virtual simulations, and treatment planning (Table 5) [61–67]. These models and platforms are already being utilized or showing potential for orthodontics education.

**Table 1:** The study characters of LLMs in orthodontics

Study setting		Methods			Outcome	
Study	Country	Questions/Exam	Answers	Evaluators	Results	Validated Utility
Tanaka 2023 [15]	Brazil	45 questions on Clear aligners, Temporary anchorage devices and Digital imaging in orthodontics	ChatGPT-4	3 orthodontists over 20-year and 2 PhD orthodontic students	4.9% were considered as very poor, 1.8% as poor, 6.7% as acceptable, 15.1% as good, and 71.6% as very good	Clinical / Educational Utility
Abu Arqub 2024 [16]	USA	111 queries on clear aligner treatment	ChatGPT	5 orthodontists and Clear aligner treatment experts	58% were true, 18% partially accurate, 9% minimally accurate, and 15% false.	Clinical Utility
Alkhamees 2024 [17]	Saudi Arabia	60 questions on impacted canines, interceptive orthodontic treatment, and orthognathic surgery	ChatGPT-4	5 orthodontists	High-quality responses (3.89/5) across all topics; inter-rater agreement among experts was low	Clinical Utility
Arslan 2024 [18]	Turkey	24 questions on conventional braces, clear aligners, orthognathic surgery, and orthodontic retainers	ChatGPT, Google Bard	An orthodontist and an orthodontic resident	Both provided satisfactory answers, with ChatGPT slightly outperforming Bard; Moderate correlation with cited references but not with word count; Significant different ratingswere observed between orthodontist and an orthodontic resident.	Clinical / Educational Utility
Daraqel 2024 [19]	China	100 FAQ including age-specific considerations for orthodontic treatment, clear aligners, combined orthodontic-orthognathic surgery treatment, digital orthodontics, expansion, extraction-based treatment, impacted teeth, mini-implants, orthodontic treatment information, and retention	ChatGPT-3.5, Google Bard	5 orthodontics	The accuracy score was (9/10) for ChatGPT and (8/10) for Google Bard. The odds of accuracy and completeness were higher in ChatGPT(31%) than in Google Bard(23%). Google Bard’s response generation time was significantly shorter than that of ChatGPT by 10.4 s/question. The response lengthgeneration and completeness score was similar in both models.	Clinical Utility
Dursun 2024 [20]	Turkey	20 most FAQ on clear aligners	ChatGPT-3.5,ChatGPT-4, Gemini, and Copilot	2 orthodontics	In terms of reliability and quality, Copilot had a higher score than other models. Gemini’s score was higher than ChatGPT-3.5. Gemini also had a significantly higher score in readability compared to both ChatGPT-4, Copilot and ChatGPT-3.5.	Clinical Utility
Hatia 2024 [21]	Italy	21 clinical open-ended questions encompassing 7 areas of interceptive orthodontics and 7 comprehensive clinical cases	ChatGPT-4	10 orthodontics	For the open-ended questions, the median score was (4.9/6) for the accuracy and (2.4/3) for completeness. For the clinical cases, the median score was (4.9/6) for accuracy and (2.5/3) for completeness.	Clinical Utility
Jaworski 2024 [22]	Poland	200 multiple-choice questions from Polish Final Dentistry Examination database of the Center for Medical Education including Conservative Dentistry with Endodontics, Pediatric Dentistry, Dental Surgery, Prosthetic Dentistry, Periodontology, Orthodontics, Emergency Medicine, Bioethics and Medical Law, Medical Certification, and Public Health	ChatGPT-4o	The correct answers from database of the Center for Medical Education	GPT-4o correctly answered 70.85% questions, performed better in fields like Conservative Dentistry with Endodontics (71.74%) and Prosthetic Dentistry (80%) but showed lower accuracy in Pediatric Dentistry (62.07%) and Orthodontics (52.63%).The accuracy of clinical case-based questions was only 36.36% while of other factual questions was 72.87%.	Educational Utility
Kılınç 2024 [23]	Turkey	34 questions about orthodontics including general questions and treatment-related questions	ChatGPT’s March 23 version and May 24 version on April 20, 2023, and July 12, 2023	2 orthodontics	In both evaluations, the reliability of the answers was found to be moderate.Grade Level results were found at the college graduate level for general questions and at the high school level for treatment-related questions. In the second evaluation, the reliability of the answers increased.The mean Reading Ease score decreased.	Clinical / Educational Utility
Kurt 2024 [24]	Turkey	Questions related to four fundamental areas of orthodontics: clear aligners, lingual orthodontics, esthetic braces, temporomandibular disorders	ChatGPT4	35 orthodontists, 35 fifth-year dental students, 35 individuals aged 18 years and older who expressed a desire for orthodontic treatment	The patients rated the ChatGPT responses highest, followed by senior dental students and orthodontists.The DISCERN scale (assessing reliability) and GQS scores(overall quality) showed the same trend	Clinical / Educational Utility
Makrygiannakis 2024 [25]	Greece	10 open-type clinical orthodontics-related questions	Google Bard, ChatGPT-3.5, ChatGPT-4, and Microsoft Bing	2 orthodontics involved in undergraduate and postgraduate teaching of orthodontics	Microsoft Bing Chat scored the highest (7.1/10), followed by ChatGPT-4 (4.7/10), Google Bard (4.6/10), and finally ChatGPT 3.5 (3.8/10). While Microsoft Bing Chat statistically outperformed ChatGPT-3.5 and Google Bard, as well, and ChatGPT-4 outperformed ChatGPT-3.5	Clinical / Educational Utility
Morishita 2024 [26]	Japan	160 questions from the 116th Japan National Dental Examination, consisting of three areas: compulsory questions, general questions, and clinical practical questions	ChatGPT-4 V	Answers from the question database of Japan National Dental Examination	The overall correct response rate was 35.0%, with 57.1% for compulsory questions, 43.6% for general questions, and 28.6% for clinical practical questions. In specialties like Orthodontics, the correct response rate was below 25%.	Educational Utility
Naureen 2024 [27]	Pakistan	90 questions about mini implant-assisted rapid palatal expansion, clear aligners, and cone beam computed tomography (CBCT), sourced from recent review articles	ChatGPT-4, Google Bard	2 independent raters with postgraduate qualifications in orthodontics	GPT-4 outperformed Google Bard in providing up-to-date information on current orthodontic treatment trends, achieving a total score of 92.6% versus Bard’s 72%.	Clinical Utility
Vassis 2024 [28]	Danmark	Orthodontic treatment side effects using self-formulated and standardized prompts by 28 patients	ChatGPT-3.5, ChatGPT-4	3 experts (two orthodontists with more than ten-year work experience & one postgraduate student); 46 patients (standard text from the postgraduate orthodontic program at Aarhus University)	Experts rated AI content as “neither deficient nor satisfactory,” with GPT-4 outperforming GPT-3.5. Patients found AI-generated information more useful, comprehensive, and reassuring, with nearly 80% preferring it over the standard text	Clinical / Educational Utility
Abuabara 2025 [29]	Brazil	Demirjian scores of left permanent mandibular tooth from panoramic radiographs	GPT-4-turbo, Gemini 2.0 Flash, DeepSeek-V3	The chronological age	All can estimate dental age from Demirjian scores, but falls short of traditional methods. DeepSeek showed consistent performance and superior accuracy compared to ChatGPT and Gemini. Gemini had the highest variability, while ChatGPT and DeepSeek showed higher errors, with Gemini performing in between.	Clinical Utility
Babaee 2025 [30]	Iran	70 dental cases spanning endodontics, oral and maxillofacial surgery, oral medicine, orthodontics, paediatric dentistry, periodontology, and radiology	ChatGPT-3.5, ChatGPT-4	Specialty-specific expert panels (two primary assessors and one adjudicator each specialty)	In scenario-based orthodontic tasks, GPT-4 produced higher-quality responses than GPT-3.5 (Good: 60% vs. 30%)	Clinical / Educational Utility
Chen 2025 [31]	China	50 most common pre-treatment consultation questions from adult orthodontic patients	Ernie Bot, ChatGPT, and Gemini	4 experts with master’s degrees in orthodontics, four adult patients	Most scores falling in the 3–4 point range and few achieving high quality (5 points)	Clinical Utility
Erdem 2025 [32]	Turkey	40 FAQ related to orthodontic emergencies, including fixed orthodontic treatment, clear aligner treatment, eating and oral hygiene, pain and discomfort, general concerns, retention, and sports and travel	ChatGPT-3.5, ChatGPT-4, Copilot, and Gemini	3 orthodontic experts (with 8, 12, and 20 years of orthodontic experience)	Gemini and ChatGPT-4.0 were most accurate, followed by Copilot, with ChatGPT-3.5 lowest. Gemini outperformed others in “Fixed Orthodontic Treatment”	Clinical Utility
Fan 2025 [33]	China	20 FAQ about the potential risks of orthodontics	ChatGPT-4o, Claude 3.5 Sonnet, and Gemini 1.5 Pro	5 orthodontists	In both low- and high-threshold validity tests, Gemini showed the highest performance, though no significant differences were observed among the three. All chatbots demonstrated satisfactory reliability, with Gemini having the highest consistency.	Clinical Utility
Fukuda 2025 [34]	Japan	400 Japan National Dental Examination (JNDTE) questions, consisting of nine main categories: dental technology and dental care, structure and function of the teeth and oral cavity, dental materials, dental laboratory equipment, and processing technology, cervical denture technical engineering, restorative dental technology, orthodontic technical engineering, and paediatric dental technology	ChatGPT-4o	2 dental educators, the official answer keys provided in the JNDTE Booklet	The AI performed well on basic knowledge questions but struggled with specialized topics requiring technical skills, particularly in “Orthodontic technology” (46.2%). Accuracy decreased further when visual elements like radiographs, figures, and procedural images were involved.	Educational Utility
Hassan 2025 [35]	USA	45 TMD - related inquiries	ChatGPT-3.5, ChatGPT-4o, Google Gemini	3 reviewers who has over 10 years of experience in clinical orthodontics	Google Gemini was most accurate (100%), while ChatGPT-4o provided the most complete (91.1%) and reliable (93.3%) responses. References appeared in ChatGPT-4o (22.2%) and Google Gemini (13.3%), with multimedia only in Google Gemini (6.7%). Overall, ChatGPT-4o and Google Gemini delivered clear, reliable answers with specialist safeguards, but lacked comprehensive evidence-based references.	Clinical / Educational Utility
Hatipoğlu 2025 [36]	Turkey	115 surgically-assisted rapid palatal expansion-related questions	ChatGPT-4, Gemini, Copilot	3 orthodontists and 1 oral and maxillofacial surgeon	Responses across AI models were generally similar. ChatGPT-4 had the highest true response rate, Gemini showed more balanced accuracy, and Copilot produced the most false answers	Clinical Utility
Hou 2025 [37]	USA	72 text-based items and 26 image-based questions from Dental Admission Test (DAT)	General models (e.g., GPT-3.5, GPT-4, GPT-4o, GPT-o1, Google’s Bard, mistral-large, and Claude), domain-specific fine- tuned models (e.g., DentalGPT, MedGPT, and BioGPT), and open-source models (e.g., Llama2-7B, Llama2- 13B, Llama2-70B, Llama3-8B, and Llama3-70B)	2 subject matter experts	GPT-4o and GPT-o1 excelled in text-based knowledge and comprehension, with GPT-o1 achieving perfect scores in natural sciences and reading comprehension. Llama3-70B performed competitively in reading tasks. All models struggled substantially with perceptual ability (PA) items, highlighting a persistent limitation in handling image-based tasks requiring visual-spatial reasoning	Educational Utility
Martina 2025 [38]	Italy	8-item yes/no orthodontic questionnaire	ChatGPT-4, Claude-3-Opus, Gemini 2.0 Flash Experimental, Microsoft Copilot, and DeepSeek	284 general practitioners and 174 orthodontists	All showed high consistency, yet most answers differed significantly from general practitioners and orthodontists responses. DeepSeek had the highest educational value by Global Quality Scale, Copilot the lowest.	Clinical Utility
Metin 2025 [39]	Turkey	40 true and false orthodontic questionnaires	ChatGPT-4, ChatGPT-4o, Microsoft Copilot, Google Gemini 1.5 Pro, and Claude 3.5 Sonnet	Clinicians (30 dental students, 30 general dentists, and 30 orthodontists)	ChatGPT-4o’s accuracy was closest to orthodontists; other AIs outperformed students and dentists but were less accurate than orthodontists. ChatGPT-4 gave the most consistent answers, whereas ChatGPT-4o and Claude 3.5 Sonnet showed the least consistency.	Clinical / Educational Utility
Mine 2025 [40]	Japan	353 questions from the 2024 Japanese National Dental Examination, including 204 text-only and 149 visually-based questions	ChatGPT-4o, OpenAI o1, Claude 3.5 Sonnet, and Gemini 2.0 Flash Thinking Experimental	Explanatory Guide for the 117th National Dental Examination Questions published by Azabu Dental Academy (Tokyo, Japan)	o1 achieved the highest overall correct response rate (81.9%), followed by Sonnet (71.7%), Gemini (66.6%), and 4o (65.7%). All models performed significantly better on text-only questions (79.9–92.2%) than on visually-based questions (45.6–67.8%). Performance varied by specialty, with highest scores in basic medical sciences (Dental pharmacology: 100%; Oral physiology: 86.7–100%) and lower scores in clinical specialties requiring visual interpretation (Orthodontics: 36.4–66.7%).	Educational Utility
Patil 2025 [41]	USA	150 multiple-choice questions on orthodontic questions from the National Board of Dental Examiners examinations	ChatGPT-4, ChatGPT 4o, Google Gemini, Microsoft CoPilot	National Board of Dental Examiners examinations	Microsoft CoPilot demonstrated the highest reliability, while ChatGPT-4.0 had the highest accuracy, but performance varied across trials. ChatGPT 4o, though newer, proved less reliable than ChatGPT 4.0.	Educational Utility
Salmanpour 2025 [42]	Turkey	Fifteen questions were randomly selected from the American Association of Orthodontists FAQ, covering orthodontic treatments, patient care, and post-treatment guidelines	ChatGPT-4, Microsoft Copilot, experts	52 orthodontists and 102 patients	ChatGPT-4.0 showed strong performance—scoring highest on questions about loose brackets or wires—but still trailed expert responses in several areas. Microsoft Copilot scored lowest overall, though it performed similarly to other models on certain topics, such as orthodontic pain and retainers.	Clinical Utility
Sismanoglu 2025 [43]	Turkey	Dental specialization exam (DUS) administered in Turkey for 2020 (118 questions) and 2021 (120 questions), including in basic medicine and clinical dentistry	ChatGPT-4, Gemini	Answers from question database of Turkish dental specialization exam; The year’s best performers	ChatGPT-4.0 achieved higher accuracy than Gemini Advanced (83.3% vs. 65% in 2020; 80.5% vs. 60.2% in 2021, *p* < 0.05). Both models scored below the top human performers (2020: 65.5 and 50.1 vs. 68.5; 2021: 65.6 and 48.6 vs. 72.3). Lower performance was also noted in basic and clinical sciences, with orthodontics showing the poorest results.	Educational Utility
Song 2025 [44]	Korea	1401 text-based Korean National Dental Licensing Examination from 2019 to 2023 covering 13 clinical subjects	GPT-4o, Gemini Advanced	Student examinees (2019—2023)	Orthodontic Subject-wise accuracy: GPT-4o 71.34%, Gemini Advanced 68.51%. Average yearly accuracy: GPT-4o 81.1%, Gemini Advanced 76.59%, students 95%. Accuracy on self-generated questions: GPT-4o 92.5%, Gemini Advanced 95%.	Educational Utility
Tanaka 2025 [45]	USA	Clinical case treated by an experienced orthodontist with over three decades of experience	ChatGPT, Google Bard, Microsoft Bing	The GenAI-generated plans, along with the orthodontist’s plan, were evaluated by 34 orthodontists	Orthodontists had significantly higher agreement with their own treatment plans than those generated by AI platforms. VAS scores for agreement: orthodontists 78.38/100, ChatGPT 46.94/100, Google Bard 40.29/100, and Microsoft Bing 47.09/100. Likert scale scores: orthodontists 4.41/5, ChatGPT 3.09/5, Google Bard 2.79/5, and Microsoft Bing 3.24/5.	Clinical Utility
Tassoker 2025 [46]	Turkey	74 text-based anatomy questions in the Turkish Dental Specialty Admission Exam between 2012 and 2021	ChatGPT- 4o, DeepSeek-v3, Gemini 2.0, and Claude 3.7 Sonnet	Answers published on the official website	Mean response time: ChatGPT-4o 10.56s, DeepSeek-v3 24.25s, Gemini 2.0 4.47s, Claude 3.7 Sonnet 9.28s; accuracy: ChatGPT-4o 98.6%, DeepSeek-v3 89.2%, Gemini 2.0 89.2%, Claude 3.7 Sonnet 89.2%	Educational Utility
Tunca 2025 [47]	Turkey	40 frequently asked orthodontic questions at three time points	ChatGPT-3.5, ChatGPT-4.0, Gemini, and Gemini-Advanced	2 orthodontic experts	Inter-rater agreement was substantial, repeatability ranged from 0.666 (Gemini) to 0.960 (ChatGPT-3.5). ChatGPT-3.5 differed significantly from Gemini and Gemini Advanced, and correlations over time were weak.	Clinical Utility
Wafaie 2025 [48]	Egypt	120 intraoral occlusal images	ChatGPT-4o mini, Microsoft Copilot, and Claude 3.5 Sonne	2 orthodontists	Claude 3.5 Sonnet successfully classified the severity of dental crowding in 50% of the images, followed by ChatGPT-4o mini (44%), and Copilot (34%). Claude and ChatGPT-4o mini had higher visual recognition rates (99%) compared to Copilot (72%). ChatGPT-4o mini had significantly slower response times. Repeated analyses showed improvement in image classification for both ChatGPT-4o mini and Copilot, while Claude 3.5 Sonnet misclassified a significant portion of the images	Clinical Utility
Xiong 2025 [49]	China	200 questions from Chinese Dental Licensing Examination	Qwen2-72b, ChatGLM4, ERNIE 4.0 Turbo, Doubao-pro 32k, XFW XingHuo 4.0 Turbo, ChatGPT, GPT-4	Answers from Chinese Dental Licensing Examination	Most models passed the Chinese Dental Licensing Examination, with Doubao-pro 32k and Qwen2-72b achieving highest accuracy (81%). Doubao-pro 32k highest 98% agreement. Doubao-pro 32k outperformed GPT-4 in answering and explaining questions, delivering effective instructional content.	Educational Utility
Yousuf 2025 [50]	Pakistan	125 questions about 3D-printed orthodontic appliances	ChatGPT-4, Copilot	7 orthodontist	Moderate-strong negative correlation between accuracy and completeness for both chatbots. ChatGPT-4 (92.4% as “objectively true’’ and 53.33% as “fully comprehensive’’) outperformed Microsoft Copilot (56.19% as “objectively true’’ and 12.38% as “fully comprehensive”)	Clinical Utility
Zhou 2025 [51]	China	30 representative orthodontic FAQs	ChatGPT-4 vs. 2 orthodontists	4 senior orthodontic experts	ChatGPT was ranked first in 50.8%, second in 29.2%, and third in 20.0%. The mean rank of ChatGPT was significantly better than that of Orthodontists	Clinical Utility
Zhu 2025 [52]	China	Dental Bench: DentalQA (the first bilingual high-quality QA dataset for dentistry) and DentalCorpus (a large-scale domain-specific English-Chinese corpus)	General (GPT-4o, GPT-4o-mini, Deepseek-V3, Deepseek-R1, Llama-3.2-3B, Llama-3.1-8B, Qwen2.5-1.5B, Qwen2.5-3B, Qwen2.5-7B, Qwen2.5-14B, Qwen2.5-32B), Medical (BioMistral-7B, HuatuoGPT2-7B, Llama-3-8B-UltraMedical), Domain Adaptation on Qwen2.5-3B (Qwen2.5-3B, w. SFT, w. RAG, w. SFT + RAG)	DentalBench Dataset	DeepSeek-R1 achieves state-of-the-art accuracy on both single-answer and multi-answer multiple choice questions, with DeepSeek-V3 and Qwen2.5-32B close behind. Conversely, on DentalBench-EN, GPT-4o leads across these tasks. Further experiments with Qwen-2.5-3B demonstrate that domain adaptation substantially improves model performance, particularly on knowledge-intensive and terminology-focused tasks.	Clinical / Educational Utility

**Table 2:** The technical performance of LLMs in orthodontics

Model	Developer	Accuracy	Reliability	Readability	Completeness	Speed
GPT-3.5	OpenAI	Temporomandibular Joint Disorders (High) Orthodontic Plan (Moderate) Clear Aligners (Moderate) Orthodontic Emergencies (Moderate) Orthodontic Side Effects (Low) General Orthodontic Questions (High) Open-Ended Clinical Reasoning orthodontic tasks (Low) Chinese Dental Licensing Examination (Moderate)	Temporomandibular Joint Disorders (Low) Orthodontic Plan (Low) Orthognathic Surgery (Moderate) Conventional Braces (Moderate) Clear Aligners (Low-Moderate) Orthodontic Retainers (Moderate) Orthodontic Side Effects (Low) Frequently Asked Orthodontic Questions (High)	Temporomandibular Joint Disorders (Low) Orthodontist plan (Low) Orthodontic clear aligners (Low)	Temporomandibular Joint Disorders (Moderate) General orthodontic questions (Moderate)	Dental Crowding Assessment (15s) General orthodontic Questions (16.2s)
GPT-4	OpenAI	Impacted Canines (Moderate) Temporomandibular Disorders (Moderate) CBCT and Digital Image (High) Interceptive Orthodontic Treatment (Moderate) Orthognathic Surgery (Moderate) Clear Aligners (High) Esthetic Orthodontic Braces (Moderate) Lingual Orthodontics (Moderate) 3D-Printed Orthodontic Appliances (High) Temporary Anchorage Devices (High) Mini Implant-Assisted Rapid Palatal Expansion (High) Orthodontic Side Effects (Low) Orthodontic Emergencies (High) Common Orthodontic Questions (Moderate) True and False Orthodontic Questions (High) Multiple-Choice Orthodontic Questions from National Board of Dental Examinations (Moderate) Open-Ended Clinical Reasoning Orthodontic Tasks (Modetrate) Dental Age Estimation (Low) Turkish Dental Specialization Exam on Orthodontics (Moderate) Chinese Dental Licensing Examination (Moderate)	Impacted Canines (Low) Temporomandibular Disorders (Moderate) Interceptive Orthodontic Treatment (Low-Moderate) Orthognathic Surgery (Low) Clear Aligners (Low-Moderate) Lingual Orthodontics (Moderate) Esthetic Orthodontic Braces (Moderate) Orthodontic Side Effects (Low) Frequently Asked Orthodontic Questions (Moderate) True and False Orthodontic Questionnaires (Low) Multiple-Choice Orthodontic Questions from the National Board of Dental Examinations (Moderate)	Clear Aligners (Low) Surgically-Assisted Rapid Palatal Expansion-Related Questions (Moderate) True and False Orthodontic Questions (High)	Clinical Cases on Interceptive Orthodontics (Moderate) 3D-printed Orthodontic Appliances (Low) Open-ended Questions on Interceptive Orthodontics (Moderate)	3D-printed Orthodontic Appliances (1.87s)
GPT-4 V	OpenAI	Image-based Japanese National Dental Examination Orthodontic Questions(Low)	N/A	N/A	N/A	N/A
GPT-4o	OpenAI	Temporomandibular Joint Disorders (High) Orthodontic Technical Engineering (Low) Orthodontic Risks (Moderate) Dental Crowding Assessment (Low) True and false orthodontic questions (High) Text-based Korean National Dental Licensing Examination on Orthodontics (Moderate) Text-based Anatomy Questions in Turkish Dental Specialty Admission Exam (High) DentalQA-ZH (Moderate) DentalQA-EN (Moderate) Text-based knowledge and comprehension in DAT (High) Polish Final Dentistry Examination on Orthodontics (Moderate) Japanese National Dental Examination Orthodontics Specialty on text-only questions (Moderate) and on visually-based questions (Low) Multiple-choice Orthodontic Questions from National Board of Dental examinations (Moderate)	Temporomandibular Joint Disorders (High) True and False Orthodontic Questions (Low) Multiple-choice Questions on Orthodontic Questions from the National Board of Dental Examiners Examinations (Moderate)	Temporomandibular Joint Disorders (Low)	Temporomandibular Joint Disorders (High)	Text-based Anatomy Questions in Turkish Dental Specialty Admission Exam (10.56s)
GPT-o1	OpenAI	Japanese National Dental Examination Orthodontics Specialty on text-only questions (High) and on visually-based questions (Moderate) Text-based knowledge and comprehension in DAT (High)	N/A	N/A	N/A	N/A
Google Bard	Google (DeepMind)	General Orthodontic Questions (Moderate) Open-Type Clinical Orthodontics-Related Questions (Low) Mini Implant-Assisted Rapid Palatal Expansion (Moderate) Clear Aligners (Moderate) CBCT (Moderate) Orthodontic Plan (Moderate)	Orthodontic Plan (Low) Orthognathic Surgery (Moderate) Conventional Braces (Moderate) Clear Aligners (Moderate) Orthodontic Retainers (Moderate)	Orthodontist Plan (Low)	General Orthodontic Questions (High)	General Orthodontic Questions (5.8s)
Google Gemini	Google (DeepMind)	Temporomandibular Joint Disorders (High) Clear Aligners (High) Orthodontic Emergencies (High) Multiple-Choice Questions on Orthodontic Questions from the National Board of Dental Examiners Examinations (Moderate)	Temporomandibular Joint Disorders (Low) Clear Aligners (Moderate) Frequently Asked Orthodontic Questions (Moderate) Multiple-choice Questions on Orthodontic Questions from the National Board of Dental Examiners Examinations (Low-Moderate)	Temporomandibular Joint Disorders (Low) Clear Aligners (Moderate) Surgically-Assisted Rapid Palatal Expansion-Related Questions (Moderate)	Temporomandibular Joint Disorders (Low)	N/A
Gemini Advanced	Google (DeepMind)	Turkish Dental Specialization Exam on Orthodontics (Low) Text-based Korean National Dental Licensing Examination on Orthodontics (Moderate-High)	Frequently Asked Orthodontic Questions (Moderate)	N/A	N/A	N/A
Gemini 1.5 Pro	Google (DeepMind)	Orthodontic Risks (Moderate-High)	N/A	True and False Orthodontic Questions (Low)	N/A	N/A
Gemini 2.0	Google (DeepMind)	Dental Age Estimation (Moderate) Japanese National Dental Examination Orthodontics Specialty on text-only questions (Moderate) and on visually-based questions (Low) Text-based Anatomy Questions in Turkish Dental Specialty Admission Exam (High)	N/A	N/A	N/A	Text-based Anatomy Questions in Turkish Dental Specialty Admission Exam (4.47s)
Claude 3.5 Sonnet	Anthropic	Dental Crowding Assessment (Moderate) Orthodontic Risks (Moderate) True and False Orthodontic Questions (High) Japanese National Dental Examination Orthodontics Specialty on Text-Only Questions (Moderate) and on Visually-Based Questions (Low)	True and false orthodontic questionnaires (Low)	True and false orthodontic questionnaires (Low)	3D-printed orthodontic appliances (Low)	Dental Crowding Assessment (4s)
Claude 3.7 Sonnet	Anthropic	Text-based Anatomy Questions in Turkish Dental Specialty Admission Exam (High)	N/A	N/A	N/A	Text-based Anatomy Questions in Turkish Dental Specialty Admission Exam (9.28s)
Microsoft Copilot	Microsoft	Dental Crowding Assessment (Low) 3D-Printed Orthodontic Appliances (Moderate) Clear Aligners (High) Orthodontic Emergencies (Moderate) True and False Orthodontic Questions (High) Multiple-Choice Questions on Orthodontic Questions from the National Board of Dental Examiners Examinations (Moderate) Common Orthodontic Questions (Moderate)	Clear Aligners (Moderate) Multiple-Choice Questions on Orthodontic Questions from the National Board of Dental Examiners Examinations (Moderate)	Clear Aligners (Low) Surgically-Assisted Rapid Palatal Expansion-Related Questions (Moderate) True and False Orthodontic Questions (Low)	3D-printed orthodontic appliances (Low)	3D-printed Orthodontic Appliances (1.90s) \Assessment (4s)
Bing Chat	Microsoft	Orthodontic Plan (Moderate) Open-type Clinical Orthodontics-Related Questions (Moderate)	Orthodontic Plan (Low)	Orthodontic Plan (Low)	N/A	N/A
DeepSeek	DeepSeek	True and False Frequently Asked Orthodontic Questions (High)	N/A	N/A	N/A	N/A
DeepSeek-V3	DeepSeek	Dental Age Estimation (Low) Text-based Anatomy Questions in Turkish Dental Specialty Admission Exam (High) DentalQA-ZH (Moderate) DentalQA-EN (Moderate)	N/A	N/A	N/A	Text-based Anatomy Questions in Turkish Dental Specialty Admission Exam (24.25s)
DeepSeek-R1	DeepSeek	DentalQA-ZH (Moderate) DentalQA-EN (Moderate)	N/A		N/A	N/A
Ernie Bot	Baidu	Common Pre-treatment Consultation Questions (Moderate) Chinese Dental Licensing Examination (Moderate)	N/A	N/A	N/A	N/A
Qwen	Alibaba	Chinese Dental Licensing Examination (Moderate) DentalQA-ZH (Moderate) DentalQA-EN (Low)	N/A	N/A	N/A	N/A

*N/A* Not applicable

High: 85% − 100%

Moderate: 50% − 84%

Low: 0% − 49%

**Table 3:** The study characters of generative vision models in orthodontics

Study setting		Methods			Outcome	
Study	Country	Architecture	InPut	Generate	Results	Validated Utility
Doan 2021 [53]	France	CycleGAN and StarGANv2	Images without an appliance	Images contain teeth wearing aligners	StarGANv2 is more resource-efficient, supporting up to 1024 × 1024 resolution, while CycleGAN is limited to 320 × 320. Future work could focus on reducing inference time for faster, real-time output.	Clinical Utility
Chen 2022 [54]	USA	OrthoAligner(StyleGAN), CycleGAN, Contrastive Learning, HiSD, InterFaceGAN, Sefa, iOrthoPredictor(TSynNet)	Original portrait image with unaligned teeth	The edited portrait image with aligned teeth	OrthoAligner predicts orthodontic outcomes without 3D scans. Using Fréchet Inception Distance to compare the distribution of generated and real data, it achieves a score of 9.19, outperforming iOrthoPredictor (9.97), CycleGAN (11.65), Contrastive Learning (10.67), HiSD (13.82), and Sefa (10.84), but slightly trailing behind InterFaceGAN (8.92).	Clinical Utility
Chen 2023 [55]	China	TSynNet, Contour-guided pSpGAN, Semantic-guided pSpGAN	Frontal photo before orthodontic treatment	Frontal photo after orthodontic treatment	Both pSpGAN (with multi-level style features) significantly improves image quality than TSynNet. Semantic-guided pSpGAN further improves the contrast between different teeth and results in sharper boundaries, making the generated images more realistic and accurate.	Clinical Utility
Tian 2023 [56]	Singapore	Novel area-preserving GAN	Smiling and non-smiling facial images	Dental-preserving de-identified facial images	Novel area-preserving GAN effectively achieved realistic de-identification, while simultaneously maintaining a high degree of realism in the preserved dental features - including teeth, jaw shape, and lips.	Clinical / Educational Utility
Fan 2024 [57]	China	TANet, MDMs, DDIMs	Initial tooth model and the target aligned tooth model	Tooth Alignment Series Illustrating Orthodontic Motion	DDIMs achieves 0.36 mm at 5 mm error, surpassing TANet (3.24 mm), and MDMs (0.78 mm)	Clinical Utility
Lei 2024 [58]	China	DPMs, TANet, PSTN, TAligNet	Initial dental model	Predicted aligned dental model	DPMs achieves 84.7% cosine similarity accuracy, surpassing TANet (81.3%), PSTN (78.1%), and TAligNet (78.5%). It transforms malocclusion to normal occlusion, addressing alignment issues (e.g., crowding, diastema) and occlusion problems (e.g., deep overbite, overjet).	Clinical Utility
Gong 2025 [59]	China	Soft-P-CGAN, Pix2Pix, Cycle-GAN, CGAN	Lateral cephalometric radiographs pre-treatment	Predicted Lateral cephalometric radiographs post-treatment	Soft-P-CGAN achieves 98% multi-scale structural similarity—surpassing Pix2Pix (82%), Cycle-GAN (88%), and CGAN (79%)—with an average mean radial error of 1.08 mm and detection rates of 88.8%, 95.1%, 97.8%, and 100% at 2.0, 2.5, 3.0, and 4.0 mm thresholds	Clinical Utility
Kavousinejad 2025 [60]	Iran	WPGGAN-GP	Very low-resolution facial profile images (4 × 4 pixels)	High-resolution facial profile images	High-resolution images (1024 × 1024 pixels). No significant difference was found between real and generated images, with the Sliced Wasserstein Distance between them being 0.026	Clinical Utility

**Table 4:** The technical performance of generative vision models in orthodontics

Model	Application Scenario	Technical Performance
CycleGAN	Generate aligner visuals/aligned teeth on portraits; Predict lateral cephalometric radiographs post-treatment	320 × 320 resolution, Fréchet Inception Distance 11.65; 88% multi-scale structural similarity
StarGANv2	Generate aligner visuals on portraits	1024 × 1024 resolution
StyleGAN	Generate aligned teeth on portraits	Fréchet Inception Distance 9.19
HiSD	Generate aligned teeth on portraits	Fréchet Inception Distance 13.82
InterFaceGAN	Generate aligned teeth on portraits	Fréchet Inception Distance 8.92
SeFa	Generate aligned teeth on portraits	Fréchet Inception Distance 10.84
TSynNet	Generate aligned teeth on (or smiling) portraits	Fréchet Inception Distance 9.97-11.343
Contour-guided pSpGAN	Generate aligned teeth on smiling portraits	Fréchet Inception Distance 7.292
Semantic-guided pSpGAN	Generate aligned teeth on smiling portraits	Fréchet Inception Distance 6.501
Novel area-preserving GAN	Preserve dental features during facial image de-identification	1024 × 1024 resolution
Pix2Pix	Predict lateral cephalometric radiographs post-treatment	82% multi-scale structural similarity
CGAN	Predict lateral cephalometric radiographs post-treatment	79% multi-scale structural similarity
Soft-P-CGAN	Predict lateral cephalometric radiographs post-treatment	98% multi-scale structural similarity
WPGGAN-GP	Generate high-resolution facial profiles from low-resolution input	1024 × 1024 resolution
TANet	Generate tooth alignment from initial (and target aligned) model	81.3% cosine similarity accuracy; 3.24 mm at 5-mm error
PSTN	Generate tooth alignment from initial model	78.1% cosine similarity accuracy
TAligNet	Generate tooth alignment from initial model	78.5% cosine similarity accuracy
DPMs	Generate tooth alignment from initial model	84.7% cosine similarity accuracy
DDIMs	Generate tooth alignment from initial and target aligned model	0.36 mm at 5-mm error
MDMs	Generate tooth alignment from initial and target aligned model	0.78 mm at 5-mm error

**Table 5:** The orthodontic-specific platform

Study setting		Methods			Outcome	
Study	Country	Platform/Architecture	InPut	Generate	Capabilities	Validated Utility
Yang 2020 [61]	China	iOrthoPredictor: TSynNet	a frontal image of misaligned teeth and a 3D teeth model	a simulated facial frontal image with aligned teeth	iOrthoPredictor takes a frontal image of a patient with misaligned teeth and a 3D teeth model, generating a simulated facial image with aligned teeth, mimicking orthodontic treatment results	Clinical Utility
Ma 2023 [62]	China	Cephgpt-4: MiniGPT-4 and VisualGLM	cephalometric images	the cephalometric dataset and generated diagnostic reports with visual question answering	A multimodal orthodontic dataset was created with cephalometric images and doctor-patient dialogues, analyzed using U-net for landmark detection and report generation. Fine-tuned on MiniGPT-4 and VisualGLM, CephGPT-4 showed strong performance in interpreting cephalometric X-rays, offering potential to improve automated orthodontic diagnostics and treatments	Clinical Utility
Ye 2023 [63]	China	MyOrthoX, Angelalign, Digiden	Lateral cephalograms	Cephalometric landmarks	All three achieved detection rates over 85% with a 2 mm threshold, and Angelalign surpassed 78.08% at 1.0 mm	Clinical Utility
Bor 2024 [64]	Turkey	WebCeph, WeDoCeph, CephX, NemoCeph	Lateral cephalograms	Cephalometric landmarks	WebCeph and WeDoCeph had significant differences in angular and linear measurements in several skeletal classes, especially in Class II and III. CephX exhibited the most reliable and consistent results, with no significant differences in repeated measurements. NemoCeph showed the most variability in results, particularly for linear parameters like Co-A and Co-Gn	Clinical Utility
Wang 2024 [65]	China	DeepOrtho	3D tooth meshes from patients with malocclusion	simulate 3D orthodontic treatment progress	DeepOrtho iteratively denoises tooth positions until clinically optimal, outperforming prior methods in accuracy and feasibility	Clinical Utility
Xu 2024 [66]	China	TeethDreamer (diffusion model)	Five intraoral photos	high-quality 3D teeth models	A 3D-aware feature attention mechanism and a geometry-aware normal loss were incorporated into the reverse diffusion process, enabling TeethDreamer to outperform existing approaches and facilitate effective remote monitoring of orthodontic treatment	Clinical Utility
Xu 2025 [67]	China	iOrtho 11.0	Lateral cephalograms	Cephalometric landmarks	Students’ use of iOrtho 11.0 to analyze cephalometric images resulted in higher measurement accuracy and shorter measurement time compared to traditional methods.	Educational Utility

### Large language models

Large language models (LLMs), the most representative and widely applied type of GenAI, employ deep neural networks trained on massive textual corpora to perform natural language processing tasks such as text generation, question answering, and summarization [68]. LLMs, such as ChatGPT, Google Bard (rebranded as Google Gemini in 2024), and Bing Chat (renamed Microsoft Copilot in 2024), have demonstrated potential across various fields, including medical and dental/orthodontic education [7, 69]. The performance of these models may vary across different studies due to factors such as sample population, sample size, language, model version, content focus, evaluator differences, and potential biases (Table 1). In the following sections, we will introduce these models in the order of their series and updates, or according to their progressive logic, highlighting their advantages and disadvantages with appropriate comparative discussion. In addition, we have also summarized and compared the accuracy, reliability, readability, completeness, and speed of these LLMs in the Table 2.

#### OpenAI series

ChatGPT, developed by OpenAI, is based on the GPT architecture and has evolved from GPT-3 in 2020, through GPT-3.5 in 2022, to GPT-4 in 2023. GPT-3.5’s responses to orthodontic questions were moderately reliable, demonstrating a college graduate-level understanding for general questions and a high school-level understanding for treatment-related querie [23]. Regarding queries on clear aligner, the accuracy was similarly suboptimal: 58% correct, 18% partially accurate, 9% minimally accurate, and 15% incorrect [16]. GPT-4 has shown improvements over GPT-3.5 in understanding complex contexts, reasoning, multimodal capabilities (e.g., image analysis), and multilingual support. GPT-4 outperformed orthodontists in addressing orthodontic patients’ FAQ (70.8%–80%), even in non-English languages [51]. Recent studies indicate that ChatGPT-4’s answers to 45 questions on clear aligners, temporary anchorage devices, and digital imaging were rated as good (15.1%) or very good (71.6%) [15]. ChatGPT-4 also delivers high-quality answers (3.89/5) on topics such as impacted canines, interceptive orthodontic treatment, and orthognathic surgery [17], and scores (47.4 ~ 45.90/75) on clear aligners and temporomandibular disorders, as well as (3.63 ~ 3.50/5) on lingual orthodontics and esthetic braces [24]. Furthermore, GPT-4 demonstrated higher-quality responses than GPT-3.5 in handling open-ended clinical questions and scenario cases (Good: 60% vs. 30%) [30], with answers being fully accurate in 40–46% of cases and fully complete in 50–54% [21], indicating that the accuracy decreases for these complex issues.

ChatGPT-4 V, which incorporates the image recognition capabilities of ChatGPT-4, was evaluated using image-based questions from the Japanese National Dental Examination (JNDE). The overall accuracy rate was 35%, with specialties such as Orthodontics exhibiting a correct response rate below 25% [26]. The image recognition feature showed limitations, especially when handling complex, image-intensive clinical questions, indicating that it is not yet fully suitable as an educational tool for dental students [26]. Further refinement and evaluation with a larger dataset are recommended [26].

The newer version, GPT-4o, with “o” standing for “Omni,” highlights its ability to process text, images, audio, and video, surpassing existing models. ChatGPT-4o’s answers to true and false orthodontic questions were the closest in accuracy to those of an orthodontist, but have the poorest consistency in its responses [39]. Nevertheless, when it comes to reasoning tasks, GPT-4’s accuracy is not as good as that of an orthodontist. In the Polish Final Dentistry Examination (LDEK), ChatGPT-4o scored 52.63% in Orthodontics, revealing a significant disparity between clinical case-based questions (36.36%) and factual questions (72.87%) [22]. While it serves as a useful educational tool, its limited clinical reasoning highlights the gap with human expertise, excelling in factual recall but lacking critical thinking and clinical judgment [22]. ChatGPT-4o was also evaluated on the Japanese National Dental Technician Examination (JNDTE), performing well on basic knowledge but struggling with specialized topics requiring technical skills, particularly in Orthodontics (46.2%) [34]. Its accuracy further declined when handling visual content such as radiographs and images [34]. Although promising for JNDTE-related knowledge, these limitations in visual and technical content underscore the need for advancements in image recognition and simulation-based dental education [34].

OpenAI then incorporated Chain of Thought with reinforcement learning, introduced GPT-o1 which improved its reasoning capabilities [70]. On the USA Dental Admission Test (DAT), GPT-o1 scored perfectly in Natural Sciences and Reading Comprehension but struggled with Perceptual Ability tasks, highlighting limitations in visual-spatial reasoning [37]. Both GPT-o1 and GPT-4o excelled in text-based knowledge and comprehension but had difficulty with perceptual tasks [37]. In the Japanese DAT, GPT-o1 outperformed GPT-4o, achieving 81.9% correct responses compared to 65.7%, and similarly excelled in orthodontics with 66.7% accuracy versus 45.5% for GPT-4o [40].

Orthodontists rated the ChatGPT-generated content as “neither deficient nor satisfactory,” found it more approachable than standardized text [28]. While 80% of respondents preferred ChatGPT-generated information, the prompts were still insufficient to provide fully satisfactory education [28]. Patients rated ChatGPT’s responses more favorably, whereas students and orthodontists were more critical due to stricter accuracy standards, with low agreement observed among orthodontists [17, 24]. ChatGPT cannot yet replace human clinical judgment, highlighting the need for expert oversight [21, 30]. ChatGPT shows potential for education but requires greater precision for professional use [24].

#### Google (DeepMind) series

Google Bard (rebranded as Google Gemini in 2024) responded to orthodontic questions 10.4 s faster per response than GPT-3.5, with similar completeness. However, it demonstrated lower median accuracy (8 vs. 9) and slightly lower completeness—exhibiting 23% and 31% greater odds of incompleteness—compared to ChatGPT [19]. Google Bard showed lower accuracy than ChatGPT in answering orthodontic questions (e.g., conventional braces, clear aligners, orthognathic surgery, and orthodontic retainers) [18]. Although Bard achieved 72% accuracy on questions about mini implant-assisted rapid palatal expansion, clear aligners, and cone beam computed tomography (CBCT) sourced from recent review articles, it still lagged behind GPT-4, which scored 92.6% [27].

Google Gemini, capable of processing multimodal data, delivered the most accurate responses to Frequently Asked Questions (FAQ) regarding orthodontic emergencies, covering treatments, aligners, hygiene, pain relief, retention, and sports concerns, among ChatGPT-3.5, ChatGPT-4, and Copilot [32]. When responding to FAQ about clear aligners, Gemini achieved the highest readability and outperformed ChatGPT-3.5 in both quality and readability, although its accuracy was slightly lower [20]. Similarly, for TMD-related questions, Gemini reached perfect accuracy (100%), whereas ChatGPT-4o provided more complete (91.1%) and reliable (93.3%) responses [35]. Moreover, Gemini included references in 22.2% of its responses, compared to 13.3% for ChatGPT-4o, and was the only model to incorporate multimedia elements (6.7%) [35].

Gemini Advanced, service based on Gemini, has achieved performance equivalent to ChatGPT-4 on the Turkish Dental Specialization Exam (DUS) [43] and GPT-4o on Korean National Dental Licensing Examination [44]. However, it still trails behind top human candidates, recording its lowest scores in the orthodontics section [43, 44]. Google Gemini and Gemini Advanced were compared with ChatGPT-3.5 and ChatGPT-4. ChatGPT-4 and Gemini Advanced showed higher reproducibility, highlighting the need to assess both accuracy and temporal stability in GenAI-assisted orthodontic communication [47]. Gemini 1.5 Pro, more enhanced version of Gemini, has demonstrated superior performance over GPT-4o by achieving the highest accuracy and greatest consistency on orthodontic risk questions [33]. The latest version, Gemini 2.0, answers anatomy questions in the Turkish DUS in as fast as 4.47 s, compared to ChatGPT-4’s 10.56 s, with an accuracy of 89.2%, nearly matching the highest accuracy of ChatGPT-4 at 98.6% [46]. However, the readability of Gemini responses was poor, raising concerns about the consistency of their knowledge generation [20, 47]. While they show potential as patient information tools in orthodontics, they require more evidence-based content and improved readability to be fully effective.

#### Anthropic series

Claude Sonnet is a part of the Claude developed by Anthropic. Its advanced version, Claude 3.5 Sonnet, demonstrated 85.83% accuracy in true-false orthodontic questionnaire, surpassing ChatGPT-4 (80.83%) and outperforming both students and general dentists. Nevertheless, its performance remained below that of orthodontic specialists, and Claude 3.5 also showed the least consistency among the models tested [39]. The more advanced version, Claude 3.7 Sonnet, achieves up to 89.2% accuracy on text-based anatomy questions in the Turkish DUS, coming close to the highest score of ChatGPT-4 at 98.6% [46]. However, these models show a decrease in accuracy when faced with non-text-based questions. On the 2024 Japanese National Dental Examination, which included 204 text-only questions and 149 visually based questions, Claude 3.5 Sonnet’s accuracy dropped to 71.7%, although it still outperformed Gemini (66.6%) and GPT-4o (65.7%). What’s worse, its performance in orthodontics was relatively poor at 39.4%, lower than both GPT-4o (45.5%) and o1 (66.7%) [40]. Claude 3.5 Sonnet, Microsoft Copilot, and ChatGPT-4o mini were tested on intraoral occlusal images for dental crowding classification. Claude achieved the highest accuracy (50%), followed by ChatGPT-4o mini (44%) and Copilot (34%), with Claude and ChatGPT showing higher visual recognition rates (99%) than Copilot (72%). Overall, all models differed substantially from orthodontists’ evaluations, indicating the need for further refinement in image processing [48].

#### Microsoft series

Microsoft’s Copilot, developed on the GPT-4 architecture, does not match the standards set by GPT-4 in terms of accuracy and comprehensiveness when addressing topics such as orthodontic treatments, surgically-assisted rapid palatal expansion questions, 3D-printed orthodontic appliances, patient care, and post-treatment guidelines [36, 42, 50]. That said, its performance on specific topics like orthodontic pain and retainers is on par with GPT-4 [42]. When evaluated on orthodontic clear aligners, Copilot ranked highest in reliability and quality, while GPT-4 achieved the highest accuracy, followed by Copilot, GPT-3.5, and Gemini [20]. Although these models provided accurate and moderately reliable answers, they occasionally lacked accuracy, clarity, and relevance [25], with generally low readability indicating a need for improvement [20]. In another study, Copilot had the highest reliability on National Board of Dental Examiners orthodontic questions among ChatGPT-4.0, ChatGPT-4, Google Gemini, and Microsoft Copilot [41]. The observed differences may be attributed to the larger sample size of 150 questions in the latter study [41], as well as variations in study populations and evaluation methodologies [20, 25].

Bing Chat, also developed on the GPT-4 architecture by Microsoft, scored the highest (7.1/10) in 10-question evaluation for orthodontics, followed by GPT-4 (4.7/10), Google Bard (4.6/10), and GPT-3.5 (3.8/10) [25]. However, in the comparison of generated orthodontic treatment plans, Microsoft Bing achieved the highest scores, followed by GPT-3.5 and Google Bard; however, all GenAI-generated plans were consistently rated lower than those of orthodontists [45]. Since they are designed to replicate existing responses, they may struggle when faced with atypical patient cases [45].

#### DeepSeek and other series

DeepSeek is a large language model developed by DeepSeek Company, designed as an AI assistant to help users with various tasks. ChatGPT-4, Claude-3-Opus, Gemini 2.0 Flash Experimental, Microsoft Copilot, and DeepSeek showed high consistency in answering orthodontic questions, with DeepSeek achieving the highest educational value according to the Global Quality Scale, though most answers differed from those provided by dentists and orthodontists and may be misleading on controversial topics [38]. Building on this foundation, DeepSeek-V3 achieved the best results in dental age estimation using Demirjian scores among ChatGPT (GPT-4-turbo), Gemini 2.0 Flash, and DeepSeek-V3, but their performance remains inferior to traditional methods [29]. DeepSeek-R1, enhanced with reasoning reinforcement, was tested on an English–Chinese Q&A benchmark spanning 16 dental subfields. In the English setting, GPT-4o achieved the highest accuracy (73.98%), followed by DeepSeek-V3 (68.28%), DeepSeek-R1 (60.04%), and the Qwen models (38.09–58.34%) [52]. In the Chinese setting, DeepSeek-R1 led with 76.06%, with DeepSeek-V3 and Qwen2.5-32B close behind [52]. Language-related differences arise because retrieval performance is language-dependent, underscoring the need for language-specific models and further refinement before clinical application [29, 52].

Aside from Deepseek, another prominent LLM in the Chinese context is Qwen, developed by Alibaba, along with Ernie Bot, developed by Baidu. Ernie Bot outperformed ChatGPT and Gemini in providing orthodontic pre-treatment information, excelling in areas such as information completeness, professionalism, and empathy. However, its accuracy was lower and more variable. ChatGPT excelled in personalization and relevance, while Gemini demonstrated stable performance, particularly in terms of information completeness and clarity [31]. Qwen2-72b achieved accuracy rates of 0.7 and 0.89 for multiple-choice and case analysis questions in the Chinese Dental Licensing Examination, closely matching GPT-4’s 0.7 and 0.86, and surpassing Ernie 4.0 Turbo’s 0.50 and 0.63 [49]. While these models show promise in handling open-ended queries, their clinical application requires caution, professional verification, and ongoing improvements in accuracy, completeness, and humanistic care [31].

### Generative vision models

Beyond LLMs, GenAI also includes generative vision models capable of producing images. Prominent examples, such as Generative Adversarial Networks (GANs) and Diffusion Models, have already been applied in orthodontic image generation and teeth movement simulation. In the following sections, we will examine their applications across various model architectures (Table 3). In addition, we have also summarized the technical performance of these models in the Table 4. Unlike LLMs, most models have not yet been tested in orthodontic education, but their potential remains promising.

#### Generative Adversarial Networks (GANs)

GANs are a type of generative vision model within GenAI. Many variants, such as Pix2Pix, Cycle-GAN, CGAN, Soft-P-CGAN, and conditional GANs (CGANs), can predict lateral appearance changes using cephalograms, thereby assisting in treatment planning [59]. Another variant, Style-based Generative Adversarial Networks (StyleGANs) encodes 3D geometric data unsupervised to predict treatment outcomes by simulating teeth alignment from portrait images [54]. Further, pSp framework fed into a pretrained StyleGAN generator, can predict post-treatment alignment from a single frontal image, providing interpretable visualizations and improving orthodontic planning precision [55]. Moreover, CycleGAN and StarGANv2 generate aligner visuals on portraits to visualize the appearance with aligners [53]. The Progressive Growing Generative Adversarial Network with Gradient Penalty (WPGGAN-GP) generates realistic facial profiles with anatomical and perceptual accuracy, making it valuable for orthodontic education, treatment simulation, and patient privacy protection [60].

These models, by generating images, can provide a more intuitive and vivid learning experience, holding potential in orthodontic education. However, their application may raise concerns regarding accuracy and privacy. For instance, the Pix2Pix method caused edge blurring, which hindered the observation of lesion contours. The Cycle-GAN method produced images nearly identical to the post-images, suggesting it failed to learn any orthodontic transformation features. Meanwhile, the CGAN method generated images with ghosting artifacts around the lips and chin [59]. Researchers have attempted to improve these models to address these flaws. StarGANv2 is more efficient, supporting resolutions up to 1024 × 1024 and producing highly realistic images, whereas CycleGAN is limited to 320 × 320 [53]. A novel area-preserving GAN inversion method effectively de-identifies dental patient images, preserving key features and addressing privacy concerns, while maintaining diagnostic value for orthodontic education [56].

#### Diffusion models

Diffusion Models, another class of Generative Vision Models, generate high-quality data by gradually adding and then removing noise, which offers more stable training and higher-quality outputs compared to GANs. In orthodontics, the Diffusion Models like Motion Diffusion Models (MDMs) and Denoising Diffusion Implicit Models (DDIMs) have been employed for tooth arrangement by leveraging features extracted from dental models to learn the distribution of teeth transformation matrices from malocclusion to normal occlusion [57]. This can help automatically access and understand the treatment plan from the generated series of models [57]. A diffusion probabilistic models (DPMs) generate tooth transformations from malocclusion to normal occlusion, achieving alignment with satisfactory occlusion [58]. In orthodontic education, these models can serve as valuable teaching tools, helping students visualize treatment progress and outcomes. However, they may differ from clinical practice and should be used as illustrative examples. Careful case selection and thorough review are essential.

### Orthodontic-specific platform

#### DentalGPT and Ceph series

Cephalometric tools such as CephX, WeDoCeph, WebCeph, MyOrthoX, Angelalign, and Digident can automate landmark identification and generate annotations for the efficient evaluation of malocclusion and craniofacial development [63, 64]. WebCeph tends to report higher IMPA and ANB values, possibly emphasizing upper jaw protrusion or anterior teeth prominence. NemoCeph shows higher FMA values, suggesting a more relaxed approach to vertical measurements. CephX demonstrates higher NLA values, likely due to its method of calculating anterior tooth inclination [64]. MyOrthoX, Angelalign, and Digident achieved detection rates exceeding 85% at a 2 mm threshold, with Angelalign reaching 78.08% at 1.0 mm [63]. These tools significantly improved measurement efficiency. Students using Angelalign’s iOrtho 11.0 showed higher accuracy and faster analysis than traditional methods [67]. GenAI-assisted analysis boosts precision, enhances understanding of anatomical landmarks, and improves teaching effectiveness, with strong student approval [67].

DentalGPT is a LLMs built on OpenAI’s GPT-4 technology, providing cross-domain knowledge support for dentistry, including guidance on procedures, patient care tips, treatment options, and oral health trends [71]. DentalGPT showed decent results in the Natural Sciences section with a 79% score, but it encountered difficulties in areas such as Reading Comprehension, Quantitative Reasoning, and image-based Perceptual Ability tasks (e.g., cephalometric analysis) [37]. Cephalometric tools can be integrated with LLMs. CephGPT-4 is a multimodal diagnostic system combining cephalometric images with doctor-patient dialogues. It uses U-net for landmark detection and integrates MiniGPT-4 (text) and VisualGLM (images) for automate diagnosis and report generation [62]. While promising in enhancing orthodontic diagnosis, treatment planning, and education, further validation is required to confirm its reliability [62]. These Ceph platforms can also function as educational tools, helping students improve their clinical skills and expertise through hands-on practice and real-time feedback.

#### Orthodontic vision generation and simulation platform

iOrthoPredictor is a system that predicts teeth alignment in photos. It processes a frontal image of a patient with misaligned teeth along with a 3D teeth model to generate a simulated facial image showing aligned teeth, mimicking orthodontic treatment results [61]. DeepOrtho integrates CNN, LSTM, and GAN architectures to simulate orthodontic treatments for clinical planning and training. It incrementally adjusts tooth positions until achieving the desired outcome, surpassing existing solutions in both accuracy and clinical feasibility [65]. TeethDreamer utilizes five intraoral photographs and employs a diffusion model to generate multi-view images and reconstruct high-quality 3D teeth models through neural surface reconstruction [66]. Unlike scan-dependent tools, the framework’s geometry-aware normal loss guarantees accuracy in 3D reconstructions, making it valuable for remote orthodontic monitoring as well as an educational tool.

## GenAI application strategy in orthodontic education

Orthodontic education includes theory, pre-clinical, and clinical training, but traditional methods like lectures and apprenticeships often lack hands-on practical experience, leaving students disengaged. It is difficult for students to apply theory to real patients without clinical practice when they practice skills with wax Typodont [72]. The integration of digital technology into orthodontic education has emerged as valuable complement to conventional methods [73]. The integration of GenAI with established teaching strategies and learning theories could offer innovative solutions in orthodontic education. Next, we will illustrate, with examples, how GenAI’s educational applications—such as knowledge dissemination, clinical training, assessment, and medical research—can enhance traditional teaching methods.

### The integration of GenAI with education theories

The integration of student-centered teaching strategies such as Team-Based Learning (TBL), Problem-Based Learning (PBL), Case-Based Learning (CBL), Experiential Learning (EL), and Competency-Based Education (CBE) into medical and dental education has shown significant benefits in developing critical thinking, clinical reasoning, and self-directed learning. These methods, grounded in realistic clinical scenarios, enhance problem-solving skills but also face challenges such as a lack of standardized clinical cases, the need for specialized facilitators, and substantial operational demands. GenAI holds promise in addressing these issues [11, 74].

#### CBL

GenAI can create case scenarios, medical questions, and responses, aiding clinical reasoning [75]. In dental education, GenAI supports students in diagnosing and planning treatments by presenting realistic patient cases, enhancing problem-solving and critical thinking [76].

#### PBL

PBL involves analyzing complex clinical problems, including patient histories, physical exams, and treatment options. GenAI supports this process by enabling students to explore clinical issues independently, participate in GenAI-driven case discussions, and develop critical thinking skills [77, 78]. It accelerates the generation of complex cases, making PBL content more timely and less resource-intensive, while also providing detailed feedback to help students identify knowledge gaps and enhance their learning [78].

#### EL

EL process follows a four-step cycle: Experience, Reflect, Think, and Act. GenAI enhances this cycle by providing real-time feedback and a personalized learning environment. Through virtual patient simulations, case discussions, and critical thinking exercises, it bridges the gap between theory and practice [79].

#### TBL

TBL fosters clinical reasoning and teamwork among medical students through structured group activities, quizzes, and clinical case exercises, while GenAI enhances engagement and encourages discussion. With GenAI integration, students discuss GenAI-generated cases before submitting their final answers, followed by a post-review and peer discussion, where correct answers are revealed in a large group [80].

#### CBE

GenAI applications in dental education enhance CBE through personalized feedback, objective assessments, and skill development opportunities. GenAI-guided simulations and feedback systems improve procedural accuracy, radiographic interpretation, and grading efficiency, helping students refine techniques and gain insights before patient practice [81]. GenAI-assisted CBE frameworks effectively bridge knowledge gaps and prepare students for the evolving demands of a technology-driven healthcare landscape [10].

Despite effective integration, GenAI sometimes fails to provide complete answers, omitting key details and struggling with complex topics, raising concerns about the reliability in dental education [75, 76]. In CBL and PBL, GenAI has been used to present complex clinical cases to dental students. However, students often trust GenAI’s output more than their own judgment, and group discussions in TBL fail to correct GenAI-generated misinformation [76, 80]. The lack of standardized and reliable AI-driven training makes it challenging to integrate AI with CBE [81]. Therefore, review and verification during use are necessary, underscoring the importance of teacher guidance. 

### Examples of GenAI applications in orthodontic education

**Fig. 2:**
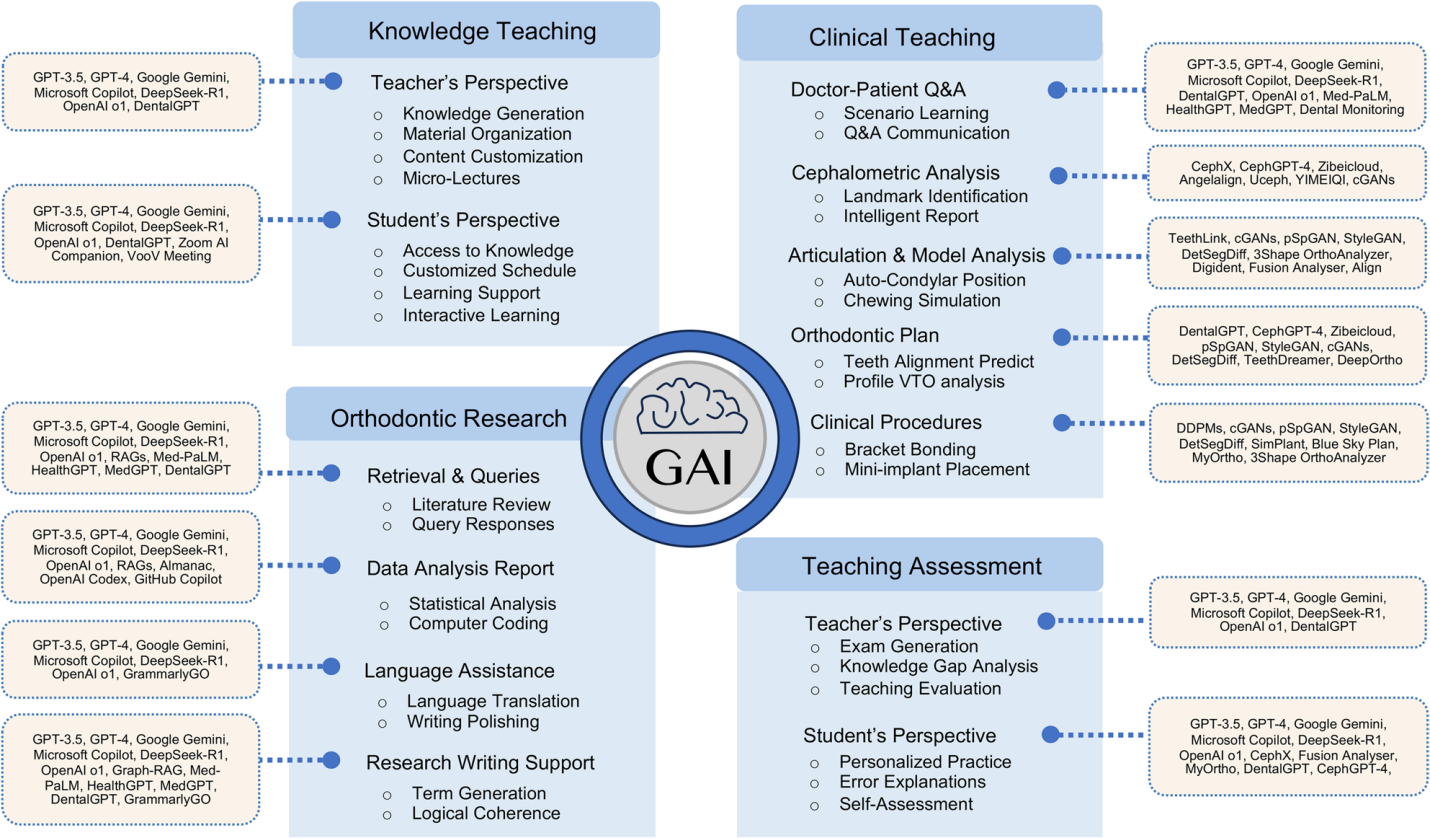
Representative GenAI application in orthodontic education

The technical capabilities of GenAI as detailed above, in conjunction with their integration with education theories, lay a groundwork for their prospective use in orthodontic education. In the subsequent sections, we will specific use examples and implementation strategies, while summarizing GenAI models/platforms that can be applicated in these domains (Fig. 2).

### Knowledge teaching

#### Teacher’s perspective

GenAI can generate clear and coherent teaching content tailored to the specific needs of orthodontic education, thereby optimizing instructional workflows. Additionally, GenAI can dynamically adapt curriculum materials by intelligently analyzing students’ learning progression, enabling the generation of more logically structured and clinically relevant teaching content (Figure S1). Moreover, teachers can leverage GenAI to create personalized teaching materials tailored to specific cases or creative needs. GenAI can generate the script for instructional videos, along with the text instructions for creating animations based on the script. These GenAI-generated instructional videos have been shown to enhance student retention, reduce cognitive load, and maintain comparable levels of satisfaction [82].

#### Student’s perspective

**Fig. 3:**
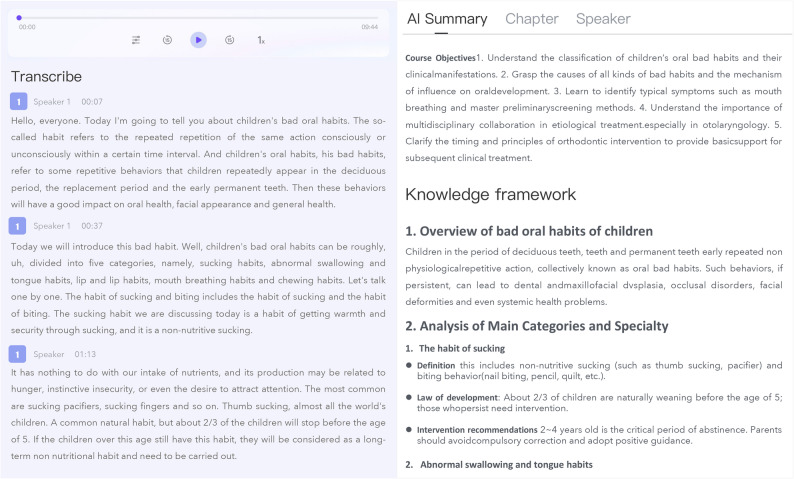
GenAI-assisted real-time translation and note-taking organization

GenAI can serve as an effective educational partner by providing personalized learning support for students. Through one-on-one simulated interactions with the GenAI chatbot, the technology enables customization of instructional content according to individual differences, subject-specific needs, and learning preferences, as well as offering intelligent tutoring and Q&A services. Additionally, GenAI can assist in organizing class content via online platforms such as Zoom AI Companion, VooV Meeting and and DingTalk Meeting. Fig. 3 illustrates the use of DingTalk Meeting (architectured by Qwen) to generate translations for online courses and assist in organizing notes.

### Clinical teaching

CBL and Technology-enhanced simulation are considered as effective for orthodontic courses [83, 84]. GenAI can simulate clinical scenarios by providing virtual cases and case analyses to help students practice clinical reasoning, decision-making, and operations, so as to develop clinical thinking abilities in a risk-free environment [48, 59]. It can automatically generate digital resources, such as 3D models and virtual scenarios, which facilitate doctor-patient communication, case studies, simulated diagnosis and treatment activities. This promotes a transition from a “teacher-centered” approach to a “student-centered” one.

#### Doctor-patient Q&A: guiding doctor-patient communication

In orthodontic education, instructors can guide students to explore the underlying mechanisms of diseases by starting with an investigation of the patient’s chief complaint and medical history, followed by an assessment of symptoms and signs, and a thorough analysis of the onset and progression of malocclusion. Students are encouraged to ask GenAI questions to deepen their understanding. For example, when a student inquires about the “causes of convex profiles” to DentalGPT, it can explain factors such as maxillary protrusion and mandibular retrognathia et al. (Figure S2), thereby helping students comprehend the symptoms and progression of malocclusion. GenAI can also offer students valuable opportunities for patient management and enhance their understanding of doctor-patient communication (Figure S3).

#### Cephalometric analysis: landmark identification and report interpretation

**Fig. 4:**
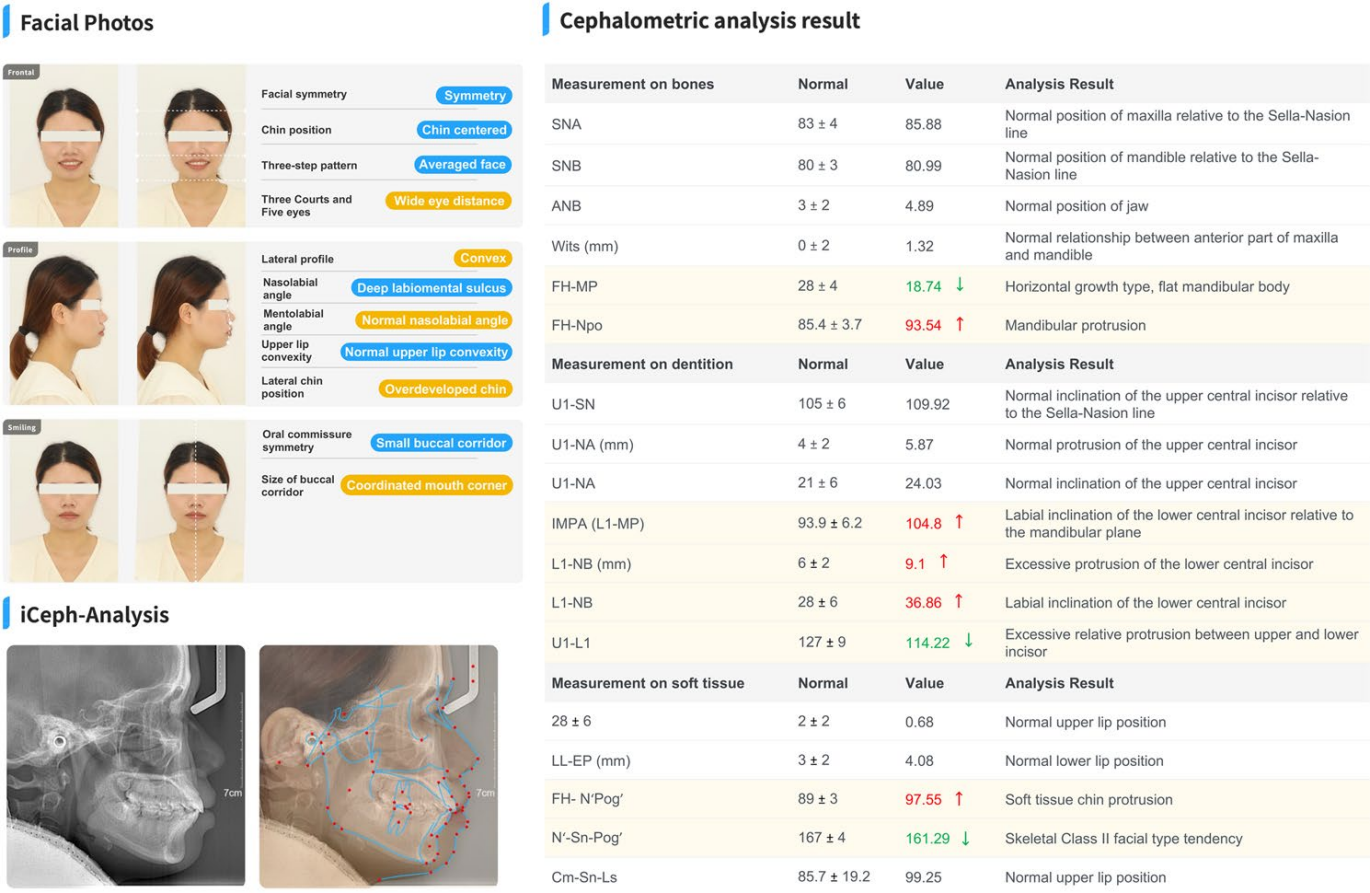
Intelligent analysis report of facial and cephalometric images

GenAI innovates the methods of orthodontic imaging data analysis, including tooth segmentation, cephalometric analysis, bone age assessment, airway analysis, panoramic bone density screening, and temporomandibular joint arthritis diagnosis, which improves diagnostic accuracy and efficiency. For example, AI-assisted cephalometric analysis increases efficiency by simplifying traditional manual and semi-automatic methods, achieving accuracy that matches or even exceeds human performance [63, 67]. Surveys indicate that 87.5% of respondents believe AI significantly reduces diagnostic time, while 72.7% feel it delivers more precise analytical results [85]. The Angelalign’s iOrtho supports students by providing semi-automatic landmark analysis and generating reports, allowing them to practice in landmark identification repeatedly. In addition, it provides single-item reports (Fig. 4) for better understanding of cephalometric items.

#### Orthodontic plan: generation of personalized medical recommendations

Integrating GenAI into the orthodontic case simulation system provides preliminary diagnoses and treatment recommendations. Research shows that AI achieves an accuracy rate of 94% in determining the necessity of teeth extraction, with accuracy rates of 84.2% and 92.8% for selecting specific extraction positions and anchorage options, respectively [86]. This can provide students with quick and relatively accurate recommendations in clinical practice. Figure S4 demonstrates Digident’s VTO analysis, visually correlating tooth angulation changes with sagittal profile alterations. Students can learn how to diagnose and treat cases in a simulated environment while receiving outcome feedback.

#### Clinical procedures: bracket positioning practice

**Fig. 5:**
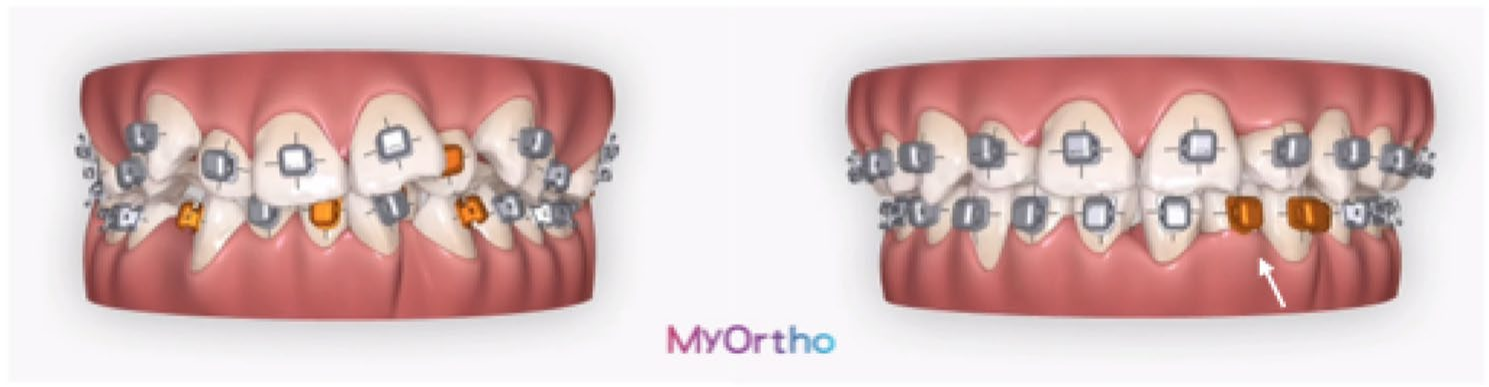
Bracket positioning training. The white arrow visually highlights tooth misalignment

Virtual Typodont, integrated with virtual reality (VR) systems, allows students to practice orthodontic procedures and simulate teeth movement in safe environments [87]. Surveys indicate that most students find VR training to be a pleasurable learning experience, with the potential to improve long-term learning outcomes [88]. The GenAI-assisted virtual simulation system can generate simulated treatment outcomes and helps students identify potential clinical risks. Systems such as 3Shape Ortho Analyzer, MyOrtho integrate virtual simulation technology with tooth alignment algorithms to facilitate bracket positioning [89]. This enable the use of bracket bonding instructions, allowing students to repeatedly practice and refine their skills in bracket placement (Fig. 5).

### Teaching assessment

GenAI technology is used to create customized assignments, exam questions, and answers. By analyzing students’ responses, teachers can identify knowledge gaps and provide targeted explanations. At the same time, students can design personalized practice exercises and mock exams tailored to their learning progress, develop test preparation strategies, and receive individualized feedback. Beyond traditional question formats, such as multiple-choice and true-or-false questions, ChatGPT can design comprehensive tasks grounded in clinical scenarios, facilitating a shift from knowledge-based Q&A to problem-solving (Figure S5). This approach provides a more holistic evaluation compared to traditional score-based assessments [90].

### Orthodontic research

In academic research, GenAI can assist with tasks such as programming, statistical analysis, literature retrieval, translation, and writing, enhancing thesis quality [9, 91]. LLMs such as ChatGPT and Google Bard can provide up-to-date information on orthodontic treatment trends [27]. ChatGPT, for instance, can generate PICO-based queries for systematic reviews [92]. GrammarlyGO is specifically designed to optimize English writing, making it a valuable tool for medical academic writing [93]. OpenAI Codex, GPT-4o and GitHub Copilot translate natural language prompts into functional code across multiple programming languages like Python, JavaScript, Go, and Ruby [94, 95]. GenAI contributes in the following areas: (1) Literature Retrieval & Queries: accelerates query responses, allowing researchers to focus on study design; (2) Data Analysis Support: assists in cephalometric analysis, statistical analysis, and code generation (Figure S6); (3) Translation & Polishing: helps non-native English researchers with technical terminology and editing; (4) Knowledge Integration & Coherence: refines ideas and ensures logical flow in writing.

## Challenges and solution in the application of GenAI

### Accuracy and reliability issues

GenAI models occasionally lacked accuracy, clarity, and relevance. While showing promise, their limitations pose risks if used without caution. They cannot replace orthodontists’ critical thinking, and further research and validation are needed for safe clinical use [25].

#### Hallucination

GenAI can generate seemingly reasonable but entirely incorrect or fabricated medical information. If students learn and internalize such erroneous knowledge, it could lead to catastrophic consequences in future clinical practice. Although certain GenAI were more accurate than students and dentists, still had a lower accuracy rate than orthodontists [39]. While somewhat informative, GenAI may provide misleading answers on controversial topics about orthodontics [38]. ChatGPT’s-False claims included CAT’s effectiveness in reducing surgery, improving airway function, and achieving root parallelism. Clear aligner treatment had the accuracy was suboptimal, and responses lacked citations. Clinicians should be cautious of false or incomplete information [16].

#### Unreliable references

The accuracy of GenAI as a source of orthodontic information depends on the level of evidence in the literature, with more consensus leading to more accurate responses [38]. However, prominent LLMs frequently provide responses to orthodontic inquiries that are not grounded in evidence-based references or outdated information [35, 42]. Uncritical use of LLMs could lead to unsafe decisions. They can complement, not replace, expert judgment and require further research, validation, and regulation [96].

#### Inconsistency

Prominent LLMs showed low repeatability on orthodontic questions, raising concerns about consistency, as they may provide different answers to the same question at different times [39, 47, 50]. Inconsistent responses pose a risk to students relying on LLMs for exam preparation or clinical decision-making [41], which is unacceptable for highly standardized medical knowledge. Notable differences were also observed in the investigators’ ratings [18]. Updates did not always improve reliability, with older models sometimes outperforming newer ones. This suggests that changes in AI model training do not always improve stability in specialized fields. The lack of agreement between models highlights the need for careful selection of GenAI tools based on professional requirements [50].

#### Poor complex clinical reasoning

Since the GenAI was developed to mimic existing answers, it may fail when encountering atypical cases [45]. Key challenges included stepwise problem-solving, knowledge transfer, complex question comprehension, arises from instances where models introduce unnecessary complexity in mathematical calculations [37]. LLMs are useful for factual learning but remain limited in higher-order reasoning and image-based tasks care [31, 52]. For example, GPT-4 and Gemini Advanced have successfully passed dental specialization exams, they still fall short of top performers in orthodontics [40, 43]. These highlight the need for further technological innovations in image recognition and simulation-based learning within dental education [26, 34, 43]. For instance, OpenAI integrated Chain of Thought with reinforcement learning to develop GPT-01, enhancing its reasoning capabilities [70].

The most effective model may be a hybrid approach—leveraging AI for scalable responses to routine inquiries while maintaining clinician oversight for more nuanced guidance [42]. Therefore, GenAI cannot fully replace orthodontists and must be used in conjunction with authoritative medical resources to ensure medical accurate for education.

### Ethical challenges

The use of GenAI in the medical field raises significant ethical concerns, particularly regarding data privacy, fairness, accountability, and doctor-patient relationships, with particular attention required when applied to teaching.

#### Privacy and security

GenAI may generate content involving sensitive medical data, such as patient histories and genetic information, posing risks to security and privacy [97]. Any data breach could result in cyberattacks and a crisis of trust between doctors and patients [98]. Privacy and ethical issues should be properly addressed to ensure the responsible integration of GenAI I into the orthodontic curriculum [11]. To mitigate privacy risks, health care organizations should implement robust security measures and privacy policy, including encryption, access controls, and regular vulnerability assessments [99]. Techniques like StyleGAN’s area-preserving method can protect patient privacy while maintaining critical teeth features [56].

#### Bias and fairness

GenAI’s rapid development can unintentionally sacrifice the interests of vulnerable groups without a legally binding ethical codex [100]. Biased algorithms can worsen health disparities, leading to unequal treatment, misdiagnoses, and poor outcomes for minority groups [101]. In clinical settings, GenAI may struggle, especially with underserved populations, resulting in errors or inappropriate care [102]. To ensure fairness, a comprehensive approach is needed—integrating diverse datasets (e.g., age, ethnicity, gender), transparent models, continuous monitoring, and cross-disciplinary collaboration [103, 104]. GANs can enhance data diversity by generating synthetic examples, improving fairness and diagnostic accuracy. A novel area-preserving GAN inversion method effectively de-identifies dental patient images (e.g., race, skin color) while preserving key diagnostic features for orthodontic education [56]. Eliminating all bias is unrealistic, but ongoing refinement and ethical evaluation are necessary [104, 105].

#### Transparency

Most LLMs prioritize accuracy over explainability, with some unable to justify their conclusions due to opaque black-box algorithms [106, 107]. Without transparency, trust and accountability are undermined, as healthcare professionals may not understand decision-making processes [108]. To improve transparency, explainability techniques can enhance LLM transparency. GenAI models can be made more interpretable through simplification or post-hoc analysis, or advanced by the Ethical Firewall Architecture built upon ethical guidelines [106–108]. While not a complete solution, these methods may lead to models with fewer flaws or at least the ability to explain their logic. For example, autoregressive and masked models can learn semantic relations with strong transparency using synthetic data [109].

#### Accountability

Determining accountability when GenAI produces harmful content is complex, involving developers, organizations, and other stakeholders. Clear legal frameworks and traceable decision-making processes are necessary to ensure responsibility [110]. Assigning responsibility requires evaluating the roles of the algorithm, training data, and user input in each case [110]. Accountability should be ensured by adding an encrypted, tamper-proof ethical core to GenAI, creating an immutable audit trail that records decisions, ethical verification, and human intervention, ensuring proper responsibility assignment [107]. Medical education should include training on the ethical and legal aspects of GenAI to ensure responsible use in teaching. Transparency and accountability can also be enhanced by proper reporting standards [106].

Utilize strategies such as “ethical firewalls”, a framework that integrates mathematically provable ethical constraints into AI decision-making. By combining formal verification, blockchain-inspired immutability, and emotion-driven escalation protocols for human oversight, it ensures that decisions are rigorously aligned with core human values prior to implementation [107].

### Excessive dependence

By offloading repetitive and time-consuming teaching tasks to GenAI, both teachers and students can focus on advanced responsibilities that require creativity, empathy, and complex judgment. However, excessive dependence on GenAI may undermine educators’ teaching capabilities and hinder students’ independence in clinical decision-making [111].

#### Impaired critical thinking

Students often tend to trust the output results of GenAI rather than their own intuition when facing a complex clinical case. Group discussions did not correct misinformation from GenAI, nor did they promote critical thinking [80]. To avoid this problem, teachers should focus on cultivating students’ critical thinking skills and fostering students’ information literacy. The emphasis should be on regulating its usage and improving assessment methods rather than imposing an outright ban to combat cheating with GenAI, especially during exams [112]. 

#### The gap in teachers’ roles

Despite GenAI tools, such as cephalometric platforms and ChatGPT-based modules, can assist in practicing diagnostic skills and gain satisfaction among students, their full adoption is hindered by limited integration, inadequate training, and faculty skepticism [9]. GenAI education struggles to replicate the rich, hands-on experience of clinical doctors and lacks the human touch essential in medical education. To address this, teachers must play a deeper role in guiding students, ensuring that the human element is not lost. In line with the “ethical firewall” strategy, human supervisors are ultimately needed to manage special situations and provide real-time monitoring [107]. Strategic reforms and faculty development are necessary to optimize GenAI’s use in orthodontic education, ensuring that it complements the teaching process.

### Academic misconduct and educational integrity

Academic misconduct and educational integrity problems are common in the use of GenAI. Some students use GenAI to complete assignments, papers, and reports, then submit the generated content directly or with minimal modifications, which undermines fairness and impairs educational integrity. Leading journals such as *Nature*, *Science*, *Cell*, *The Lancet*, and *JAMA* have introduced policies to regulate the use of GenAI [113]. AI cannot be listed as a co-author, and researchers are required to disclose AI usage and prohibit AI authorship [113]. These policies emphasize the originality, authenticity, and transparency of research outcomes, thereby ensuring the credibility of research assisted by GenAI [114]. Academic support is growing for acknowledging language learning models (LLMs) like ChatGPT. Nature’s Editor-in-Chief (Stokel-Walker) [115] suggests mentioning LLM usage in the acknowledgments section. Standard practice calls for tool descriptions and citations in the text [116].

## Conclusion and future directions

Prominent GenAI models, such as ChatGPT, DeepSeek, GANs, and Diffusion Models, offer a wide range of capabilities, from knowledge-based Q&A and clinical diagnostics to image generation and treatment simulation. While LLMs exhibit above-average accuracy in certain areas, they still fall short in critical tasks like reasoning and image-based analysis, which are essential in orthodontics and require careful oversight and validation. Generative Vision Models can simulate orthodontic treatment processes and outcomes, but they have not yet been tested in orthodontic education. Platforms like iOrtho, CephGPT-4, and iOrthoPredictor provide specialized services, including automated cephalometric analysis, virtual simulations, and treatment planning, demonstrating potential for orthodontic education. The integration of GenAI with established teaching strategies and learning theories were also introduced, with sample applications in generating content, simulating clinical scenarios, generating personalized assessments, and supporting research. Despite its promise, GenAI in orthodontics raises concerns about information accuracy, ethical, academic integrity, and over-reliance. These challenges underline the need for a deeper understanding of GenAI in clinical practice and research, as current AI systems can sometimes omit critical information, raising doubts about their reliability in medical education.

Looking ahead, future advancements in GenAI are likely to focus on multimodal models that integrate visual, speech, and language data (e.g., combining CBCT scans with verbal communication and textual diagnoses) to enhance clinical reasoning. The greatest potential lies in hybrid models, where GenAI complements mentorship, allowing faculty to focus on complex, context-dependent teaching. Additionally, GenAI-driven adaptive learning systems could personalize curricula based on real-time competency assessments, shifting education from standardized teaching to competency-based models. Currently, no studies have yet evaluated long-term learning outcomes with GenAI tutoring in orthodontics, representing an important area for future research. However, these innovations could exacerbate educational inequalities, particularly if resource-limited institutions lack access to non-open-source platforms. Addressing this challenge will require collaborative efforts to develop open-source tools and subsidize technology distribution.

In summary, while GenAI technology offers innovative opportunities for orthodontic education, it also presents challenges that necessitate careful consideration. Proper use of GenAI can enhance teaching quality and contribute to the development of orthodontic specialists.

## Supplementary Information

Supplementary Material 1.

## Data Availability

The data underlying this article will be shared on reasonable request to the corresponding author.

## Abbreviations

GenAI: Generative Artificial Intelligence
AI: Artificial Intelligence
LLMs: Large Language Models
JNDE: Japanese National Dental Examination
Q&A: Question and Answer
LDEK: Polish Final Dentistry Examination
JNDTE: Japanese National Dental Technician Examination
DAT: Dental Admission Test
CBCT: Cone beam computed tomography
DUS: Dental Specialization Exam
GANs: Generative Adversarial Networks
CGANs: Conditional Generative Adversarial Networks
StyleGAN: Style-based Generative Adversarial Networks
WPGGAN-GP: Progressive Growing GAN with Gradient Penalty
DPMs: Diffusion Probabilistic Models
DDIMs: Denoising Diffusion Implicit Models
MDMs: Motion Diffusion Models
TBL: Team-Based Learning
PBL: Problem-Based Learning
CBL: Case-Based Learning
EL: Experiential Learning
CBE: Competency-Based Education
VR: Virtual Reality
