# Supplementary material for: Generative Artificial Intelligence-driven orthodontic education practices
Source: BMC Med Educ. 2026 Apr 29;26:978. doi: 10.1186/s12909-026-09276-7 (PMC13274141; doi:10.1186/s12909-026-09276-7)
Supplement: Supplementary file 1 — Supplementary Material 1. [file 12909_2026_9276_MOESM1_ESM.docx]

**Supplementary Information**

**Table S1. The levels of evidence of the cited studies**

| Study | Q1 | Q2 | Q3 | Q4 | Q5 | Q6 | Q7 | Q8 | Q9 | Quality | Level |
| --- | --- | --- | --- | --- | --- | --- | --- | --- | --- | --- | --- |
| Tanaka 2023 | 2 | 2 | 2 | 0 | 1 | 1 | 2 | 2 | 2 | 78 | Moderate |
| Abu Arqub 2024 | 1 | 2 | 2 | 0 | 0 | 1 | 2 | 2 | 2 | 67 | Moderate |
| Alkhamees 2024 | 2 | 2 | 2 | 0 | 0 | 1 | 2 | 2 | 2 | 72 | Moderate |
| Arslan 2024 | 1 | 2 | 2 | 0 | 0 | 2 | 2 | 2 | 2 | 72 | Moderate |
| Daraqel 2024 | 2 | 2 | 2 | 2 | 2 | 2 | 2 | 2 | 1 | 94 | Strong |
| Dursun 2024 | 2 | 2 | 2 | 2 | 0 | 2 | 2 | 2 | 1 | 83 | Strong |
| Hatia 2024 | 2 | 2 | 2 | 0 | 0 | 1 | 2 | 2 | 2 | 72 | Moderate |
| Jaworski 2024 | 2 | 2 | 2 | 0 | 2 | 1 | 2 | 2 | 2 | 83 | Strong |
| Kılınç 2024 | 2 | 2 | 2 | 0 | 0 | 2 | 2 | 2 | 2 | 78 | Moderate |
| Kurt 2024 | 2 | 1 | 2 | 0 | 0 | 2 | 2 | 2 | 2 | 72 | Moderate |
| Makrygiannakis 2024 | 2 | 2 | 2 | 2 | 0 | 2 | 2 | 2 | 2 | 89 | Strong |
| Morishita 2024 | 2 | 2 | 2 | 0 | 0 | 1 | 2 | 2 | 2 | 72 | Moderate |
| Naureen 2024 | 2 | 2 | 2 | 0 | 2 | 2 | 2 | 2 | 2 | 89 | Strong |
| Vassis 2024 | 2 | 2 | 2 | 0 | 1 | 2 | 1 | 2 | 2 | 78 | Moderate |
| Abuabara 2025 | 2 | 2 | 2 | 1 | 0 | 2 | 1 | 2 | 2 | 78 | Moderate |
| Babaee 2025 | 2 | 2 | 2 | 1 | 0 | 2 | 2 | 2 | 2 | 83 | Strong |
| Chen 2025 | 2 | 2 | 2 | 0 | 1 | 2 | 1 | 2 | 2 | 78 | Moderate |
| Erdem 2025 | 2 | 2 | 2 | 2 | 0 | 2 | 2 | 2 | 2 | 89 | Strong |
| Fan 2025 | 2 | 2 | 2 | 2 | 0 | 2 | 2 | 2 | 2 | 89 | Strong |
| Fukuda 2025 | 2 | 2 | 2 | 0 | 0 | 2 | 2 | 2 | 2 | 78 | Moderate |
| Hassan 2025 | 2 | 2 | 2 | 0 | 0 | 2 | 2 | 2 | 2 | 78 | Moderate |
| Hatipoğlu 2025 | 2 | 2 | 2 | 0 | 0 | 2 | 2 | 2 | 2 | 78 | Moderate |
| Hou 2025 | 2 | 2 | 2 | 0 | 1 | 2 | 2 | 2 | 2 | 83 | Strong |
| Martina 2025 | 2 | 2 | 2 | 1 | 0 | 2 | 1 | 2 | 2 | 83 | Moderate |
| Metin 2025 | 2 | 2 | 2 | 0 | 1 | 2 | 2 | 2 | 2 | 83 | Strong |
| Mine 2025 | 2 | 2 | 2 | 0 | 0 | 2 | 2 | 2 | 2 | 78 | Moderate |
| Patil 2025 | 2 | 2 | 2 | 2 | 0 | 2 | 1 | 2 | 2 | 89 | Moderate |
| Salmanpour 2025 | 2 | 2 | 2 | 1 | 0 | 2 | 1 | 2 | 2 | 83 | Moderate |
| Sismanoglu 2025 | 2 | 2 | 2 | 0 | 0 | 2 | 2 | 2 | 2 | 78 | Moderate |
| Song 2025 | 2 | 2 | 2 | 0 | 0 | 2 | 2 | 2 | 1 | 72 | Moderate |
| Tanaka 2025 | 1 | 2 | 2 | 0 | 0 | 2 | 2 | 2 | 2 | 72 | Moderate |
| Tassoker 2025 | 2 | 2 | 2 | 0 | 0 | 2 | 2 | 2 | 1 | 72 | Moderate |
| Tunca 2025 | 2 | 2 | 2 | 2 | 0 | 2 | 2 | 2 | 1 | 83 | Strong |
| Wafaie 2025 | 2 | 2 | 2 | 0 | 2 | 2 | 1 | 2 | 2 | 78 | Moderate |
| Xiong 2025 | 2 | 2 | 2 | 0 | 0 | 2 | 2 | 2 | 1 | 72 | Moderate |
| Yousuf 2025 | 2 | 2 | 2 | 2 | 0 | 2 | 1 | 2 | 2 | 83 | Strong |
| Zhou 2025 | 2 | 2 | 2 | 0 | 1 | 2 | 2 | 2 | 1 | 78 | Moderate |
| Zhu 2025 | 2 | 2 | 2 | 0 | 1 | 2 | 2 | 2 | 2 | 83 | Strong |
| Doan 2021 | 2 | 0 | 2 | 0 | 0 | 2 | 2 | 1 | 0 | 50 | Weak |
| Chen 2022 | 2 | 2 | 2 | 0 | 1 | 2 | 2 | 2 | 1 | 78 | Moderate |
| Chen 2023 | 2 | 2 | 2 | 0 | 1 | 2 | 2 | 2 | 0 | 72 | Moderate |
| Tian 2023 | 2 | 1 | 1 | 0 | 1 | 0 | 1 | 0 | 1 | 39 | Weak |
| Fan 2024 | 2 | 1 | 2 | 0 | 1 | 0 | 1 | 0 | 0 | 38 | Weak |
| Lei 2024 | 2 | 1 | 2 | 0 | 1 | 0 | 2 | 0 | 0 | 44 | Weak |
| Gong 2025 | 2 | 2 | 2 | 0 | 1 | 2 | 2 | 2 | 0 | 72 | Moderate |
| Kavousinejad 2025 | 2 | 2 | 2 | 0 | 1 | 2 | 2 | 2 | 2 | 83 | Strong |
| Yang 2020 | 2 | 1 | 2 | 0 | 1 | 2 | 2 | 2 | 1 | 72 | Moderate |
| Ma 2023 | 2 | 1 | 0 | 0 | 0 | 0 | 1 | 0 | 1 | 28 | Weak |
| Ye 2023 | 2 | 2 | 2 | 0 | 1 | 2 | 2 | 2 | 2 | 83 | Strong |
| Bor 2024 | 2 | 2 | 2 | 0 | 2 | 2 | 2 | 2 | 2 | 89 | Strong |
| Wang 2024 | 2 | 1 | 1 | 0 | 0 | 0 | 1 | 1 | 2 | 44 | Weak |
| Xu 2024 | 2 | 2 | 2 | 0 | 1 | 2 | 2 | 2 | 1 | 78 | Moderate |
| Xu 2025 | 2 | 2 | 2 | 0 | 2 | 2 | 2 | 2 | 1 | 83 | Strong |

Q1: Is the version of GenAI model clearly described?

Q2: Is the source of the input data clearly described?

Q3: Are the evaluation methods for the output results clearly defined?

Q4: Are the uses of blinding clearly stated?

Q5: Are random methods clearly stated?

Q6: Is the experimental group design comprehensive?

Q7: Is the result reporting complete?

Q8: Are the findings validated?

Q9: Is the potential bias discussed?

Quality (%) = Score / Full Score

The overall quality: Strong (total score 80%–100%), Moderate (65%–79%), Weak (≤64%)

**Figure S1. GenAI-assisted intelligent analysis of learning progress and adaptive learning materials**

**Figure S2. The causes of convex profiles generated by GenAI**

**Figure S3. GenAI-assisted simulated doctor-patient conversation**

**Figure S4. Intelligent VTO analysis and profile prediction**

**Figure S5. Designing Comprehensive Tasks with GenAI for comprehensiveevaluation**

**Figure S6. R Code Generated for Orthodontic Clinical Research.** R code generated using GPT-4o for Mendelian randomization analysis.
